# Comparative Morphological, Metabolic and Transcriptome Analyses in *elmo1* ^*−/−*^, *elmo2* ^*−/−*^, and *elmo3* ^*−/−*^ Zebrafish Mutants Identified a Functional Non-Redundancy of the Elmo Proteins

**DOI:** 10.3389/fcell.2022.918529

**Published:** 2022-07-08

**Authors:** Mike Boger, Katrin Bennewitz, David Philipp Wohlfart, Ingrid Hausser, Carsten Sticht, Gernot Poschet, Jens Kroll

**Affiliations:** ^1^ Department of Vascular Biology and Tumor Angiogenesis, European Center for Angioscience (ECAS), Medical Faculty Mannheim, Heidelberg University, Mannheim, Germany; ^2^ Institute of Pathology IPH, EM Lab, Heidelberg University Hospital, Heidelberg, Germany; ^3^ NGS Core Facility, Medical Faculty Mannheim, Heidelberg University, Mannheim, Germany; ^4^ Metabolomics Core Technology Platform, Centre for Organismal Studies, Heidelberg University, Heidelberg, Germany

**Keywords:** ELMO1, ELMO2, ELMO3, Elmo protein family, vasculature, zebrafish

## Abstract

The ELMO protein family consists of the homologues ELMO1, ELMO2 and ELMO3. Several studies have shown that the individual ELMO proteins are involved in a variety of cellular and developmental processes. However, it has poorly been understood whether the Elmo proteins show similar functions and act redundantly. To address this question, *elmo1* ^*−/−*^, *elmo2* ^*−/−*^ and *elmo3* ^*−/−*^ zebrafish were generated and a comprehensive comparison of the phenotypic changes in organ morphology, transcriptome and metabolome was performed in these mutants. The results showed decreased fasting and increased postprandial blood glucose levels in adult *elmo1* ^*−/−*^, as well as a decreased vascular formation in the adult retina in *elmo1* ^*−/−*^, but an increased vascular formation in the adult *elmo3* ^*−/−*^ retina. The phenotypical comparison provided few similarities, as increased Bowman space areas in adult *elmo1* ^*−/−*^ and *elmo2* ^*−/−*^ kidneys, an increased hyaloid vessel diameter in *elmo1* ^*−/−*^ and *elmo3* ^*−/−*^ and a transcriptional downregulation of the vascular development in *elmo1* ^*−/−*^, *elmo2* ^*−/−*^, and *elmo3* ^*−/−*^ zebrafish larvae. Besides this, *elmo1* ^*−/−*^, *elmo2* ^*−/−*^, and *elmo3* ^*−/−*^ zebrafish exhibited several distinct changes in the vascular and glomerular structure and in the metabolome and the transcriptome. Especially, *elmo3* ^*−/−*^ zebrafish showed extensive differences in the larval transcriptome and an impaired survivability. Together, the data demonstrated that the three zebrafish Elmo proteins regulate not only similar but also divergent biological processes and mechanisms and show a low functional redundancy.

## Introduction

The engulfment and cell motility (ELMO) protein family consists of the three proteins ELMO1, ELMO2 and ELMO3. ELMO proteins are evolutionary conserved and represented in several organisms, including *C. elegans*, *D. melanogaster* and in higher vertebrates such as zebrafish, mouse and human. They are established binding partners of dedicator of cytokinesis (DOCK) family members to regulate the activity of Rac family small GTPase 1 (RAC1) through their function as guanine nucleotide exchange factors (GEF) (Gumienny et al., 2001; Zhou et al., 2001; Brugnera et al., 2002; Katoh and Negishi, 2003; Lu et al., 2004; Cetinkaya et al., 2016; Tran et al., 2021). ELMO/DOCK directed RAC1 activation regulates important basic cellular functions such as cell migration, cytoskeleton organization, phagocytosis, the engulfment of apoptotic cells and myoblast fusion (Gumienny et al., 2001; Wu et al., 2001; Brugnera et al., 2002; Grimsley et al., 2004; Park et al., 2007; Geisbrecht et al., 2008; Elliott et al., 2010; Hamoud et al., 2014; Sun et al., 2015; Peotter et al., 2016; Tran et al., 2021).

As RAC1 regulators, ELMO proteins are involved in a variety of cellular and developmental processes in which they execute specific functions, as in nervous system development and disorders (Katoh and Negishi, 2003; Biersmith et al., 2011; Franke et al., 2012; Namekata et al., 2012; Lanoue et al., 2013; Mehawej et al., 2018; Tran et al., 2021). ELMO1 and ELMO2 promote axon guidance (Makihara et al., 2018) and *elmo1* ^*−/−*^ zebrafish showed reduced apoptotic neuronal death and regulation of axon myelination and neuronal numbers (Mikdache et al., 2019). ELMO3 mutations were recently suggested as the cause of developmental delay and autism in humans (Tran et al., 2021). ELMO proteins were linked to multiple other diseases, such as cancer, diabetes mellitus, inflammatory bowel disease and arthritis (Shimazaki et al., 2005; Hathaway et al., 2016; Sayed et al., 2020; Arandjelovic et al., 2021). Respective studies provided insights into the variety of the ELMO proteins’ functions as promotive factors for cell growth, proliferation and metastasis in multiple human carcinomas (Peng et al., 2016; Hu et al., 2018; Pan et al., 2019). ELMO3 is commonly suggested as a negative prognostic marker when overexpressed in different cancer types (Fan et al., 2015; Haymerle et al., 2017; Kadletz et al., 2017). Additionally, ELMO3 was recently described as a regulator of RAC1 activity promoting cell migration (Tran et al., 2021) and thereby shown to act similar to ELMO1 and ELMO2. Further functions of ELMO3, regarding developmental processes are still poorly understood.

In mouse models, ELMO1 was identified as a microbial sensor in epithelial and phagocytic cells that activates inflammatory signals and as a required factor for bacterial internalization and monocyte recruitment in inflammatory bowel disease (Sayed et al., 2020). *Elmo1* ^*−/−*^ mouse models of osteoporosis and arthritis showed reduced bone erosion and identified ELMO1 as positive regulator of osteoclast function and bone loss (Arandjelovic et al., 2021). In addition Elmo1 was described as a promotor of angiogenesis and early vascular development in zebrafish (Epting et al., 2010; Schäker et al., 2015). Remarkably, worldwide genetic studies in humans reported a susceptibility of diabetic patients with gene variants of *ELMO1*, *ELMO2* or *ELMO3* to develop kidney damage (Shimazaki et al., 2005; Bento et al., 2008; Leak et al., 2009; Hanson et al., 2010; Wu et al., 2013; Alberto Ramirez-Garcia et al., 2015; Liu et al., 2015; Turki et al., 2018). This suggested a contribution of the *ELMO* gene family in the development of diabetic nephropathy. Studies analyzing the role of ELMO1 in kidney function described an aggravation of nephropathy in mice with type 1 diabetes (Shimazaki et al., 2005; Hathaway et al., 2016) and a promotion of glomerular injury through dysregulation of the extracellular matrix (ECM) (Shimazaki et al., 2006) in presence of ELMO1. ELMO2 was shown to regulate the insulin dependent expression and membrane translocation of GLUT4 in human skeletal muscle cells and adipocytes, indicating a potential involvement in glucose homeostasis (Sun et al., 2016).

Together, these studies have identified various functions of the individual ELMO proteins; however, most existing studies used variable experimental parameters such as different animal organisms, different cell systems, different experimental approaches and non-related research topics. Furthermore, they predominantly provided data about one ELMO protein and no comparative data about all three. Thus, it remained unclear if the three ELMO proteins share common functions and if they regulate similar or diverse biological processes.

Therefore, this study aimed to analyze the functions of all three members of the Elmo protein family in the vasculature, kidney morphology and glucose homeostasis in one animal model. We generated knockout mutant zebrafish of *elmo1*, *elmo2* and *elmo3* and identified, despite few similarities, several functional differences between *elmo1* ^*−/−*^, *elmo2* ^*−/−*^ and *elmo3* ^*−/−*^ animals. We observed an impaired survivability of *elmo3* ^*−/−*^ zebrafish, decreased fasting and increased postprandial blood glucose levels in adult *elmo1* ^*−/−*^, as well as a decreased vascular formation in the adult retina in *elmo1* ^*−/−*^ but an increased vascular formation in the adult retina in *elmo3* ^*−/−*^. Besides a common transcriptional downregulation of the vascular development in *elmo1* ^*−/−*^, *elmo2* ^*−/−*^, and *elmo3* ^*−/−*^ zebrafish larvae, the three mutants, especially *elmo3* ^*−/−*^, revealed several differences in the regulation of the larval transcriptome. These new findings clearly demonstrate a functional diversity and non-redundancy between the three Elmo proteins in zebrafish.

## Results

### Amino Acid Sequence Comparison of Elmo1, Elmo2 and Elmo3 and Generation of *elmo1* ^*−/−*^, *elmo2* ^*−/−*^ and *elmo3* ^*−/−*^ Zebrafish Lines

A comprehensive, comparative investigation, especially *in vivo*, addressing the questions if ELMO proteins share identical functions, has not been done yet. Previous studies showed overlapping and varying expression patterns of *elmo1*, *elmo2* and *elmo3* in retinal, vascular, neuronal and glomerular structures in zebrafish larvae and that Elmo1 regulates the vascular structure in the developing zebrafish and the glomerular structure in diabetic mice (Shimazaki et al., 2005; Epting et al., 2010; Cetinkaya et al., 2016; Hathaway et al., 2016). Therefore, this study aimed to analyze all three Elmo proteins in zebrafish to find potential differences and similarities.

An alignment of the amino acid sequences of zebrafish Elmo1, Elmo2 and Elmo3 showed a shared identity of 43% within all three proteins (Supplementary Figure S1). Human ELMO1, ELMO2 and ELMO3 exhibit an identity of 45% in amino acid sequence (Supplementary Figure S2), therefore similar to zebrafish. Elmo1 and Elmo2 in zebrafish displayed an identity of 63%, Elmo1 and Elmo3 58% and Elmo2 and Elmo3 50% in the amino acid sequence. The armadillo-like helical domain of unknown function present in all Elmo proteins is 41% identical, the functional not well described ELMO domain is 40% identical and the pleckstrin homology domain regulating the interaction with Dock180 to promote Rac1 activity shares 58% identity between Elmo1, Elmo2 and Elmo3 (Zhou et al., 2001; Lu et al., 2004; Bowzard et al., 2007; Komander et al., 2008; East et al., 2012). Although these amino acid comparisons showed some similarities between Elmo1, Elmo2 and Elmo3, they also exhibited remarkable differences. Therefore, zebrafish Elmo1, Elmo2 and Elmo3 are not highly conserved and variable functions between the different Elmo proteins can be expected.

**FIGURE 1:**
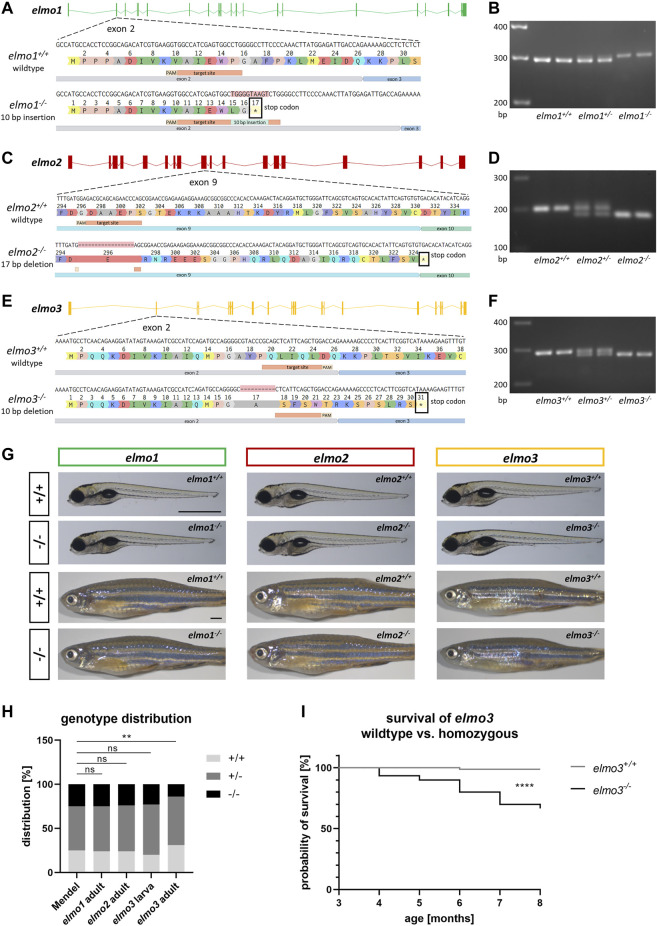
Generation of *elmo1*, *elmo2* and *elmo3* knockout zebrafish lines. **(A)** Exon map of the zebrafish *elmo1* gene and the position of the CRISPR target site designed for zebrafish *elmo1* targeting exon 2. Sequencing results of cDNA sequence and the resulting amino acid sequence from *elmo1* ^*+/+*^ and the generated *elmo1* ^*−/−*^ line with a 10 bp insertion. The resulting stop codon is indicated with a star. **(B)** The 10 base pair insertion in *elmo1* was confirmed on RNA level by gel electrophoretic segregation of PCR products of cDNA which was synthesized from mRNA from *elmo1* ^*+/+*^, *elmo1* ^*+/−*^ and *elmo1* ^*−/−*^ larvae at 120 hpf. PCR product size: wild type 298 bp, homozygous 308 bp. **(C)** Exon map of the zebrafish *elmo2* gene and the position of the CRISPR target site designed for zebrafish *elmo2* targeting exon 9. Sequencing results of cDNA sequence and the resulting amino acid sequence from *elmo2* ^*+/+*^ and the generated *elmo2* ^*−/−*^ line with a 17 bp deletion. The resulting stop codon is indicated with a star. **(D)** The 17 base pair deletion in *elmo2* was confirmed on RNA level by gel electrophoretic segregation of PCR products of cDNA which was synthesized from mRNA from *elmo2* ^*+/+*^, *elmo2* ^*+/-*^ and *elmo2* ^*−/−*^ larvae at 120 hpf. PCR product size: wild type 198 bp, homozygous 181 bp. **(E)** Exon map of the zebrafish *elmo3* gene and the position of the CRISPR target site designed for zebrafish *elmo3* targeting exon 2. Sequencing results of cDNA sequence and the resulting amino acid sequence from *elmo3* ^*+/+*^ and the generated *elmo3* ^*−/−*^ line with a 10 bp deletion. The resulting stop codon is indicated with a star. **(F)** The 10 base pair deletion in *elmo3* was confirmed on RNA level by gel electrophoretic segregation of PCR products of cDNA which was synthesized from mRNA from *elmo3* ^*+/+*^, *elmo3* ^*+/−*^, and *elmo3* ^*−/−*^ larvae at 120 hpf. PCR product size: wild type 290 bp, homozygous 280 bp. **(G)** Representative light microscopy pictures of larvae at 120 hpf and adults (8–13 mpf) of *elmo1* ^*+/+*^, *elmo1* ^*−/−*^, *elmo2* ^*+/+*^, *elmo2* ^*−/−*^, *elmo3* ^*+/+*^, and *elmo3* ^*−/−*^ zebrafish. Scale bar is 1 mm. **(H)** Genotype distribution of Mendelian inheritance and the filial generation of *elmo1* (*n* = 358), *elmo2* (*n* = 354) and *elmo3* (*n*_larva_ = 100, *n*_adult_ = 224) after heterozygous *inter se* crossings. Age of adults was 3 mpf and age of larvae was 120 hpf. The genotype distribution in *elmo3* adults was altered to the Mendelian inheritance. **(I)** Reduced survival rate of adult *elmo3* ^*−/−*^ (*n* = 30) compared to *elmo3* ^*+/+*^ (*n* = 74) zebrafish over the age of 3–8 months. Statistical analysis was done with chi-square test and logrank test. **p < 0.01, ****p < 0.0001. ns, not significant; bp, base pair; hpf, hours post fertilization; mpf, months post fertilization.

To investigate the functions of Elmo1, Elmo2 and Elmo3 through the consequences of the loss of each protein, *elmo1*, *elmo2* and *elmo3* knockout zebrafish lines were generated with the CRISPR/Cas9 system in the reporter line *Tg(fli1:EGFP)*. For each gene a frameshift mutation was generated which resulted in an early stop codon in the mRNA sequence and therefore in a premature termination of the translation (Figures 1A,C,E). For the *elmo1* ^*−/−*^ line, a 10 base pair (bp) insertion in exon 2 was induced (Figure 1A), for the *elmo2* ^*−/−*^ line, a 17 bp deletion in exon 9 (Figure 1C) and for the *elmo3* ^*−/−*^ line, a 10 bp deletion in exon 2 were induced (Figure 1E). The mutation in *elmo1* is predicted to lead to a 16 amino acids short version of the Elmo1 protein lacking all three known domains. The mutation in *elmo2* is predicted to lead to a 324 amino acids short version of the Elmo2 protein lacking the majority of the ELMO domain and the entire PH domain. The mutation in *elmo3* is predicted to lead to a 30 amino acids short version of the Elmo3 protein lacking all three domains. Therefore, the created premature-translation-termination codons in *elmo1* ^*−/−*^, *elmo2* ^*−/−*^ and *elmo3* ^*−/−*^ zebrafish lead to remaining peptides which cannot be functional and result in the loss of the respective protein. The mutations were analyzed and verified by sequencing genomic DNA. Furthermore, mutations were validated on RNA level by reverse transcription polymerase chain reaction (RT-PCR), for each gene, respectively (Figures 1B,D,F).

Due to the homology within the Elmo protein family in zebrafish (Supplementary Figure S1), expression analyses in zebrafish larvae were applied *via* reverse transcription quantitative polymerase chain reaction (RT-qPCR) to assess the expression of the *elmo* genes in the early development in wild types (Supplementary Figure S3A) and to examine if they compensate for each other in *elmo1* ^*−/−*^, *elmo2* ^*−/−*^ and *elmo3* ^*−/−*^ at 120 hpf, respectively (Supplementary Figure S4). In wild type zebrafish larvae the expression of *elmo1* was over ten times higher than the expression of *elmo2* and over five times higher than the expression of *elmo3* over the first 5 days of development (Supplementary Figure S3A). In *elmo1* ^*−/−*^ mutant larvae *elmo1* RNA was decreased (Supplementary Figure S4A) and in *elmo2* ^*−/−*^ mutant larvae *elmo2* RNA was decreased at 120 hpf (Supplementary Figure S4B), whereas *elmo3* RNA was not changed in *elmo3* ^*−/−*^ mutant larvae (Supplementary Figure S4C). Mutations which lead to a premature-translation-termination codon can cause transcript degradation by nonsense-mediated decay or reduced transcription through epigenetic silencing (El-Brolosy and Stainier, 2017; El-Brolosy et al., 2019; Sztal and Stainier, 2020). Therefore, the decreased RNA of *elmo1* in *elmo1* ^*−/−*^ larvae and the decreased RNA of *elmo2* in *elmo2* ^*−/−*^ larvae can be seen as an additional confirmation of the respective frameshift mutations. Likewise the mutated RNA can be insensitive, escape or not trigger this event (Denecke et al., 2004; Inácio et al., 2004; Dabrowska et al., 2018). Remarkably, *elmo1* expression was increased in *elmo2* ^*−/−*^ larvae (Supplementary Figure S4B) which could indicate a compensatory mechanism of Elmo1 during a loss of Elmo2, hinting at a functional similarity.

### Reduced Viability of *elmo3* ^*−/−*^ Zebrafish

In embryonic and larval development gross morphology and viability of *elmo1* ^*−/−*^, *elmo2* ^*−/−*^ and *elmo3* ^*−/−*^ zebrafish appeared unaltered compared to their wild type littermates until 120 hours post fertilization (hpf) (Figure 1G). Analysis of genotypes after heterozygous incrosses revealed the expected distribution equal to the Mendelian inheritance of *elmo3* larvae (120 hpf) and of adult [3 months post fertilization (mpf)] *elmo1* and *elmo2* animals (Figure 1H); however, *elmo3* adults (3 mpf) showed an altered genotype distribution with 32% *elmo3* ^*+/+*^, 55% *elmo3* ^*+/−*^ and 13% *elmo3* ^*−/−*^ animals. A subsequent analysis found a significant and progressive decrease in the survival rate of *elmo3* ^*−/−*^ zebrafish starting from 3 months after birth (Figure 1I) due to yet unknown reasons. Together, these results showed a normal development and viability of *elmo1* ^*−/−*^ and *elmo2* ^*−/−*^ zebrafish, but a reduced viability of *elmo3* ^*−/−*^ mutants.

### *elmo1* ^*−/−*^ Zebrafish Exhibited a Reduced and *elmo3* ^*−/−*^ an Increased Vascular Formation in the Adult Retina

**FIGURE 2:**
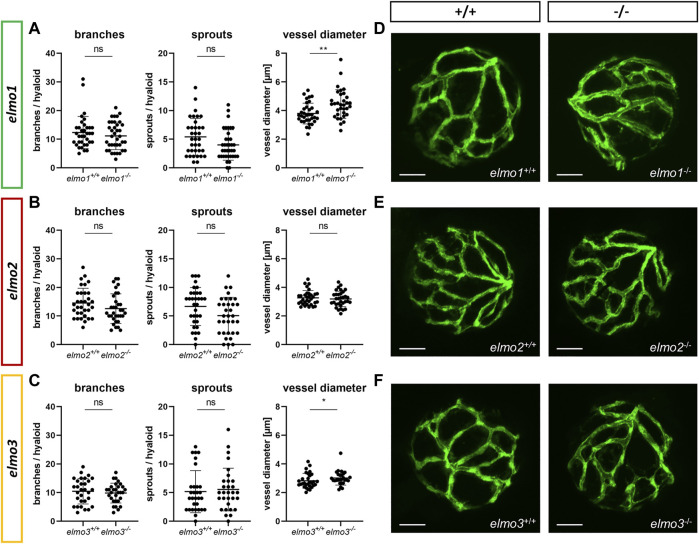
The loss of Elmo1 and Elmo3, respectively, led to an increased diameter of hyaloid blood vessels in zebrafish larvae. **(A)** Quantification of larval hyaloid vasculature at 120 hpf showed no changes in branches and sprouts but an increase in the vessel diameter in *elmo1* ^*−/−*^ compared to *elmo1* ^*+/+*^. *n* = 33–37 hyaloids per group. **(B)** Quantification of larval hyaloid vasculature at 120 hpf showed no changes in branches, sprouts and vessel diameter in *elmo2* ^*−/−*^ compared to *elmo2* ^*+/+*^. *n* = 33 hyaloids per group. **(C)** Quantification of larval hyaloid vasculature at 120 hpf showed no changes in branches and sprouts but an increase in the vessel diameter in *elmo3* ^*−/−*^ compared to *elmo3* ^*+/+*^. *n* = 27–31 hyaloids per group. **(D–F)** Representative confocal microscopy images of the hyaloid vasculature of *elmo1* ^*+/+*^ and *elmo1* ^*−/−*^ **(D)**, *elmo2* ^*+/+*^ and *elmo2* ^*−/−*^ **(E)** and *elmo3* ^*+/+*^ and *elmo3* ^*−/−*^ **(F)** zebrafish larvae at 120 hpf. Scale bar is 50 µm. Statistical analysis was done with *t*-test and Mann-Whitney test. *p < 0.05, **p < 0.01. ns, not significant; hpf, hours post fertilization.

**FIGURE 3:**
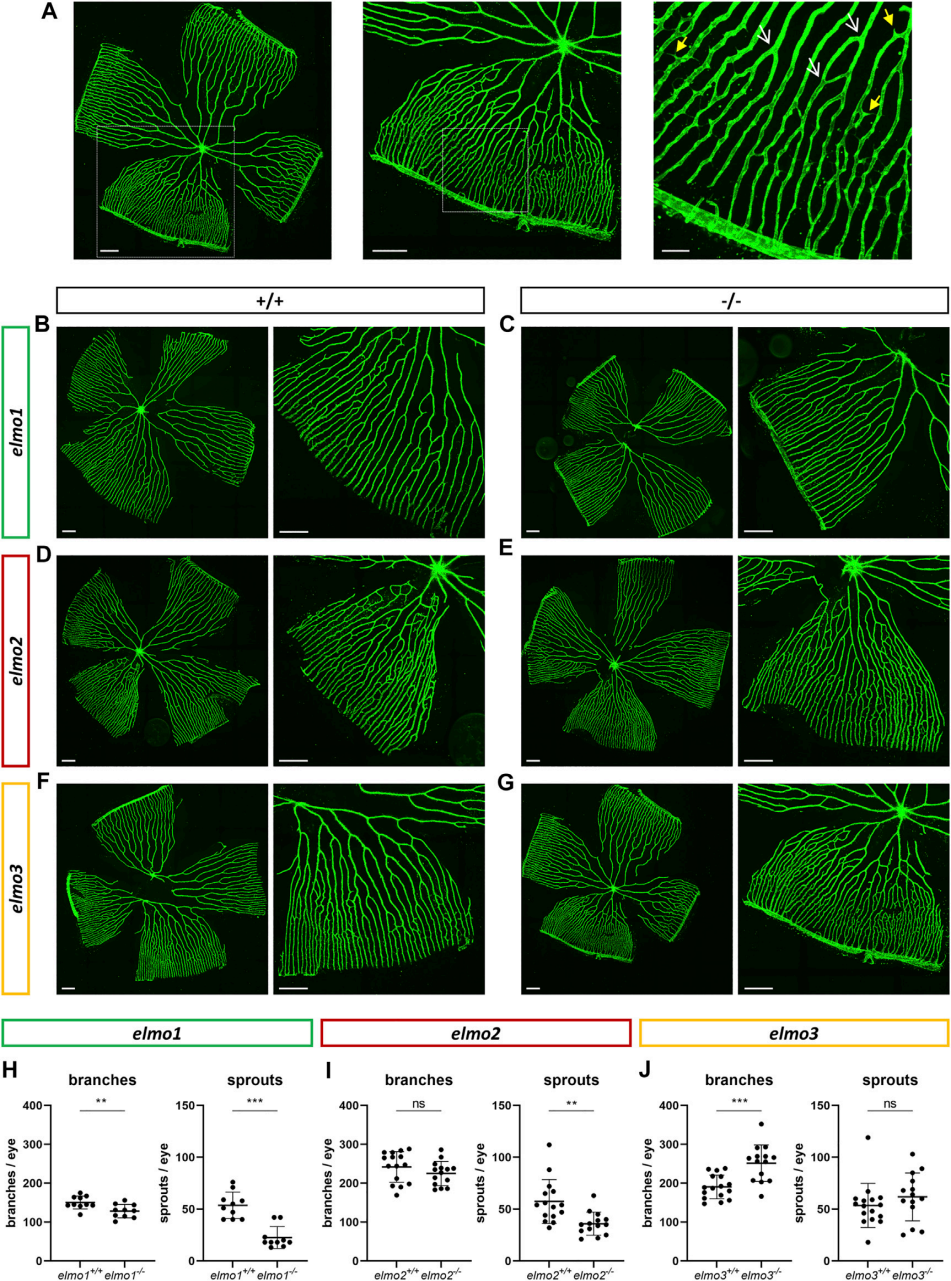
Different regulation of the retinal vasculature through Elmo1, Elmo2 and Elmo3 in adult zebrafish. **(A)** Exemplary pictures of a confocal scan of an adult zebrafish retinal vasculature. White squares each indicate the section of the image on the right. Scale bars are 200 µm (left, middle) and 50 µm (right). Representative vascular branches are indicated with a yellow arrow with a full arrowhead and sprouts are indicated with a white arrow with an open arrowhead. **(B–G)** Representative confocal microscopy images of the retina vasculature of *elmo1* ^*+/+*^ **(B)** and *elmo1* ^*−/−*^ **(C)**, *elmo2* ^*+/+*^ **(D)** and *elmo2* ^*−/−*^ **(E)** and *elmo3* ^*+/+*^ **(F)** and *elmo3* ^*−/−*^ **(G)** adult zebrafish. **(H)** Quantification of the adult retina vasculature showed a decrease in branches and sprouts in *elmo1* ^*−/−*^ compared to *elmo1* ^*+/+*^. *n* = 10 retinae per group. **(I)** Quantification of the adult retina vasculature showed no changes in branches and a decrease in sprouts in *elmo2* ^*−/−*^ compared to *elmo2* ^*+/+*^. *n* = 14–15 retinae per group. **(J)** Quantification of the adult retina vasculature showed an increase in branches and no changes in sprouts in *elmo3* ^*−/−*^ compared to *elmo3* ^*+/+*^. *n* = 14–16 retinae per group. Analyzed retinae were obtained from animals of 9–15 mpf. Scale bar is 200 µm. Statistical analysis was done with *t*-test and Mann-Whitney test. **p < 0.01, ***p < 0.001. ns, not significant; mpf, months post fertilization.

Recent reports have shown that an Elmo1 mediated Rac1 activation during angiogenesis induces and maintains the formation of the zebrafish vasculature (Epting et al., 2010; Schäker et al., 2015). Furthermore, RAC1 positively regulates angiogenesis and a loss of RAC1 led to strong vascular alterations in embryonic mice (Tan et al., 2008). To address which impact the loss of Elmo1, Elmo2 or Elmo3 may have on the vasculature in zebrafish, the larval vascular morphology of the trunk (32 and 96 hpf) (Supplementary Figures S5, S6), the larval hyaloid vasculature (120 hpf) (Figures 2A–F) and the adult retinal vasculature (9–15 mpf) (Figures 3A–J) was analyzed in *elmo1* ^*−/−*^, *elmo2* ^*−/−*^, and *elmo3* ^*−/−*^ zebrafish. The larval trunk vasculature in *elmo1* ^*−/−*^, *elmo2* ^*−/−*^ and *elmo3* ^*−/−*^ (Supplementary Figure S5) appeared mostly unaltered. Solely the number of additional sprouts between the intersegmental vessels decreased in *elmo1* ^*−/−*^ mutants compared to *elmo1* ^*+/+*^ (Supplementary Figure S5B). However, at 32 hpf *elmo1* ^*−/−*^ zebrafish larvae displayed a developmental delay of the trunk vasculature compared to *elmo1* ^*+/+*^, which was expressed in ISV formation defects and additional sprouts (Supplementary Figure S6) but not present anymore at 96 hpf. The vasculature of the hyaloids, the precursor of the retinal vasculature (Saint-Geniez and D'Amore, 2004), of *elmo1* ^*−/−*^, *elmo2* ^*−/−*^, and *elmo3* ^*−/−*^ larvae exhibited no changes in vascular branching and sprouting compared to *elmo1* ^*+/+*^, *elmo2* ^*+/+*^, and *elmo3* ^*+/+*^ (Figure 2). Interestingly, both *elmo1* ^*−/−*^ and *elmo3* ^*−/−*^ zebrafish revealed an increase in the vessel diameter (Figures 2A,C,D,F). In contrast, in the adult retinal vasculature (Figure 3A), branches and sprouts decreased in *elmo1* ^*−/−*^ zebrafish compared to *elmo1* ^*+/+*^ (Figures 3B,E). In *elmo2* ^*−/−*^ zebrafish, branches were unchanged, but sprouts also decreased compared to *elmo2* ^*+/+*^ (Figures 3C,F). Contrary to the reduced vascular formation in *elmo1* ^*−/−*^ retinae branches in *elmo3* ^*−/−*^ zebrafish increased and sprouts showed no change compared to *elmo3* ^*+/+*^ (Figures 3D,G). RT-qPCR based expression experiments on adult eyes (13 mpf) revealed a different expression of *elmo1*, *elmo2*, and *elmo3* (Supplementary Figure S3B), providing a potential explanation for the different phenotypes. Together, the data in the *elmo1* ^*−/−*^, *elmo2* ^*−/−*^, and *elmo3* ^*−/−*^ zebrafish mutants revealed different phenotypic alterations in the trunk, hyaloid and retinal vasculature, which suggests the Elmo proteins as important regulators of the vasculature modulating similar and diverse processes in vascular development and morphogenesis.

### The Loss of Elmo Proteins Led to Structural Changes in Adult Zebrafish Glomeruli

**FIGURE 4:**
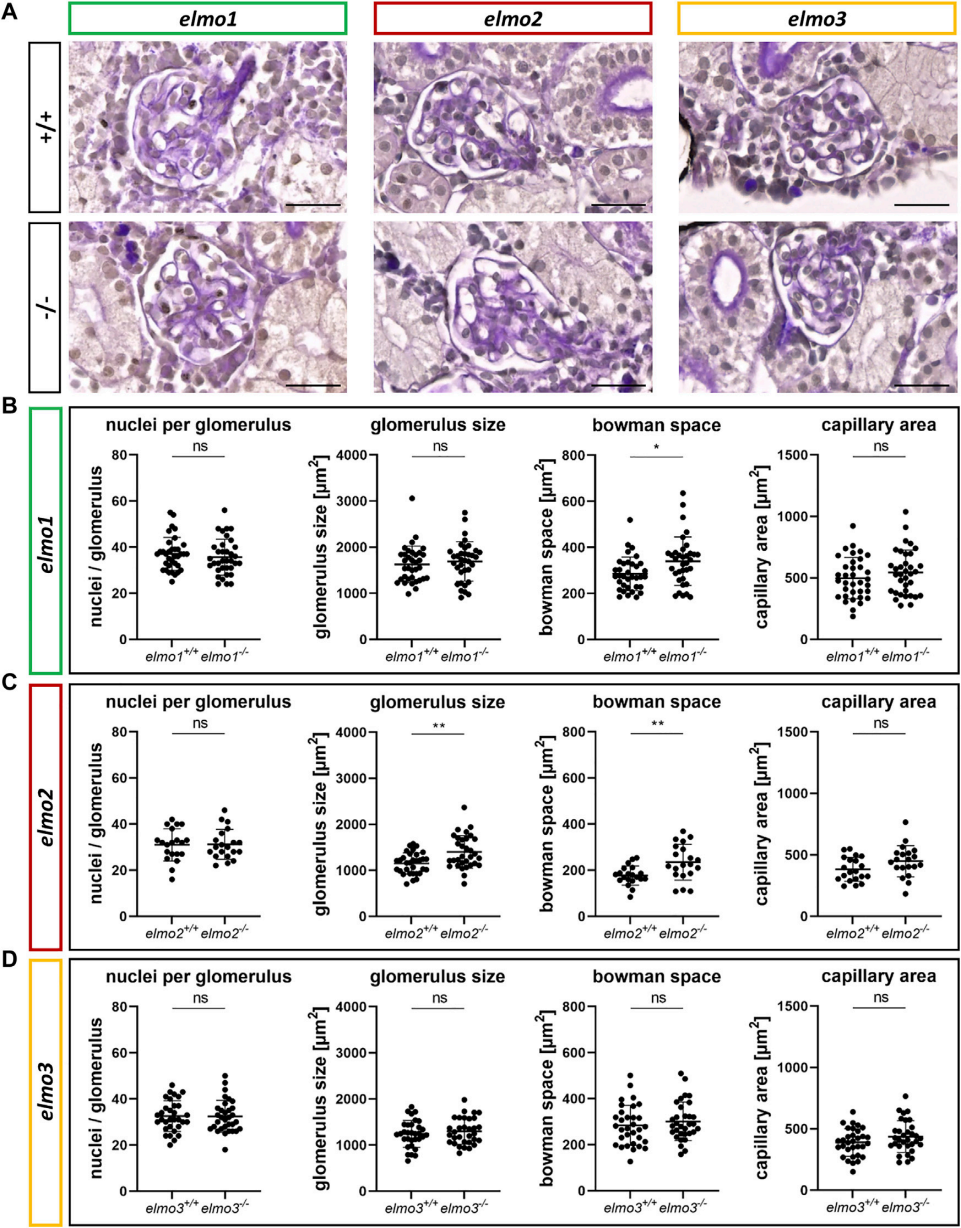
Different regulation of the glomerular structure through Elmo1, Elmo2 and Elmo3 in the kidney of adult zebrafish. **(A)** Representative light microscopy images of PAS stained sections of glomeruli in the kidney of *elmo1* ^*+/+*^ and *elmo1* ^*−/−*^, *elmo2* ^*+/+*^ and *elmo2* ^*−/−*^ and *elmo3* ^*+/+*^ and *elmo3* ^*−/−*^ adult zebrafish (9–13 mpf). Scale bar is 20 µm. **(B)** Analysis of the glomerulus structure in adult zebrafish kidneys showed no changes in the number of nuclei, glomerulus size and capillary area but an increase in the bowman space in *elmo1* ^*−/−*^ compared to *elmo1* ^*+/+*^. *n* = 35 glomeruli per group, always seven glomeruli per kidney. **(C)** Analysis of the glomerulus structure in adult zebrafish kidneys showed no changes in the number of nuclei and the capillary area but an increase in glomerulus size and bowman space in *elmo2* ^*−/−*^ compared to *elmo2* ^*+/+*^. *n* = 20 glomeruli per group, always five per kidney. **(D)** Analysis of the glomerulus structure in adult zebrafish kidneys showed no alterations in *elmo3* ^*−/−*^ compared to *elmo3* ^*+/+*^. *n* = 32 glomeruli per group, always eight per kidney. Statistical analysis was done with *t*-test and Mann-Whitney test. *p < 0.05, **p < 0.01. ns, not significant; mpf, months post fertilization.

**FIGURE 5:**
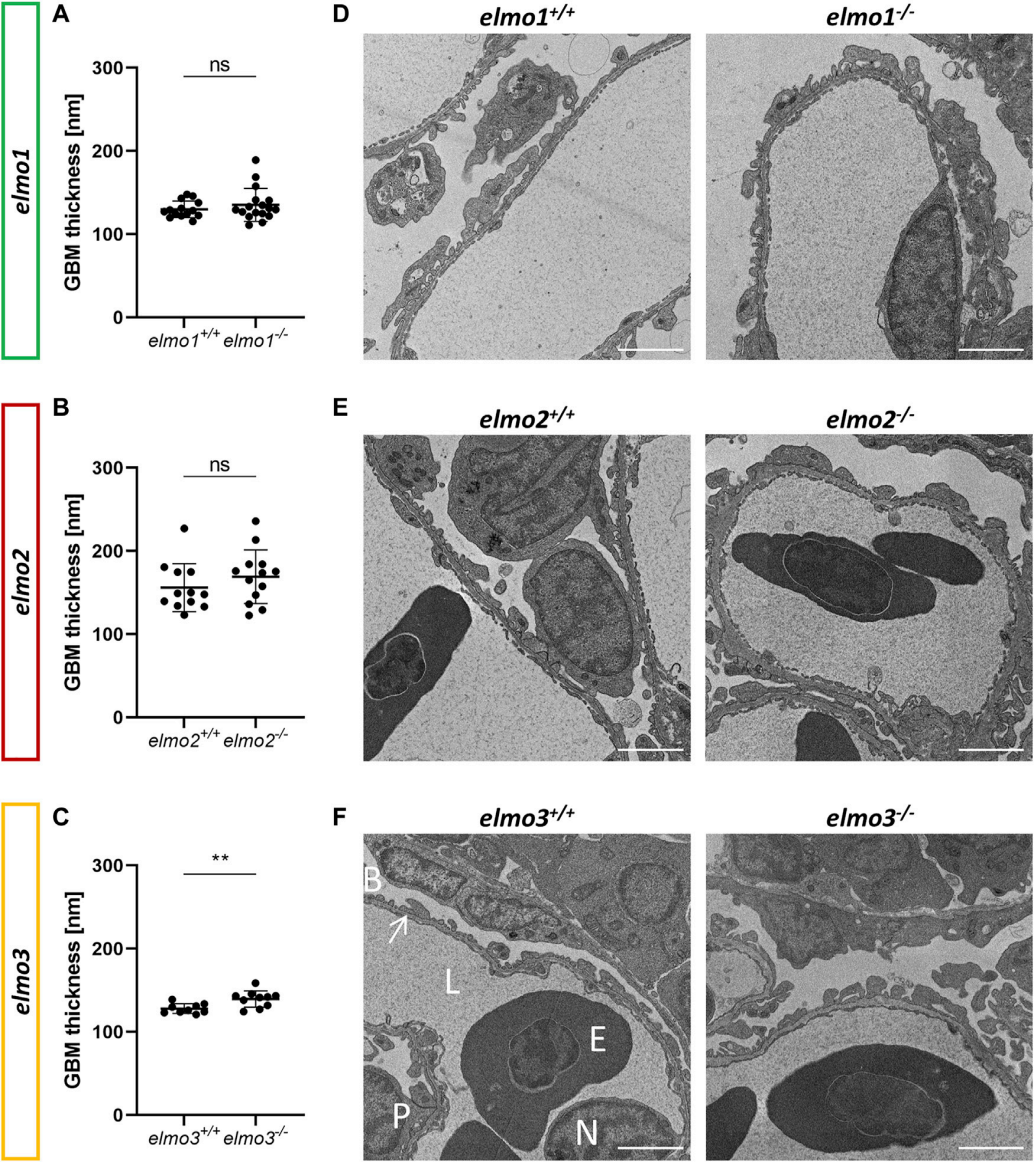
The loss of Elmo3 led to a thickening of the glomerular basement membrane in adult zebrafish kidneys. **(A)** Quantification of the thickness of the GBM in the adult kidney (9–15 mpf) showed no alterations in *elmo1* ^*−/−*^ compared to littermate *elmo1* ^*+/+*^. *n* = 14–17 per group. Each is the mean thickness per one glomerulus. **(B)** Quantification of the thickness of the GBM in the adult kidney showed no alterations in *elmo2* ^*−/−*^ compared to littermate *elmo2* ^*+/+*^. *n* = 12–13 per group. Each is the mean thickness per one glomerulus. **(C)** Quantification of the thickness of the GBM in the adult kidney showed an increase of the GBM thickness in *elmo3* ^*−/−*^ compared to littermate *elmo3* ^*+/+*^. *n* = 9–10 per group. Each is the mean thickness per one glomerulus **(D–F)** Representative electron microscopy images of glomerulus sections of adult zebrafish kidneys of *elmo1* ^*+/+*^ and *elmo1* ^*−/−*^ **(D)**, *elmo2* ^*+/+*^ and *elmo2* ^*−/−*^ **(E)** and *elmo3* ^*+/+*^ and *elmo3* ^*−/−*^ **(F)**. Exemplarily glomerulus compartments as GBM (arrow), Bowman space (B), endothelial cell nucleus (N), capillary lumen (L), podocyte (P) and erythrocyte (E) are indicated in white. Scale bar is 2 µm. Statistical analysis was done with *t*-test and Mann-Whitney test. **p < 0.01. ns, not significant; GBM, glomerular basement membrane; mpf, months post fertilization.

*ELMO* gene variants have been associated with diabetic kidney disease (Shimazaki et al., 2005; Bento et al., 2008; Leak et al., 2009; Hanson et al., 2010; Wu et al., 2013; Alberto Ramirez-Garcia et al., 2015; Liu et al., 2015; Turki et al., 2018), especially for ELMO1, which promotes the development of nephropathy in diabetic mice (Hathaway et al., 2016), and was suggested to promote the development of glomerular injury through dysregulation of the extracellular matrix (ECM) (Shimazaki et al., 2006). To investigate if the three Elmo proteins have an essential function in kidney formation, we analyzed whether adult *elmo1* ^*−/−*^, *elmo2* ^*−/−*^, and *elmo3* ^*−/−*^ zebrafish kidneys (9–15 mpf) showed an altered morphology with a special focus on the glomeruli. The *elmo* gene expression (Supplementary Figure S3B) and overall structure of the kidney and the glomerular structures were analyzed *via* PAS staining and quantified for the cell number, the size of the glomeruli and their corresponding bowman space and capillary area. The gross morphology of the kidneys showed no abnormal alterations in *elmo1* ^*−/−*^, *elmo2* ^*−/−*^, and *elmo3* ^*−/−*^ zebrafish (Figure 4). Furthermore, in *elmo1* ^*−/−*^, *elmo2* ^*−/−*^, and *elmo3* ^*−/−*^ the number of glomerular nuclei was unchanged compared to their wild type littermates (Figures 4A–D). *elmo1* ^*−/−*^ glomeruli exhibited an unchanged glomerulus size and capillary area, but an increased bowman space (Figure 4B). *elmo2* ^*−/−*^ glomeruli also displayed an increased bowman space besides an increased glomerulus size, but no change in the capillary area (Figure 4C). *elmo3* ^*−/−*^ glomeruli showed no changes in the size of the glomeruli, the bowman space or the capillary area (Figure 4D). To analyze if parts of the filtration unit of the glomerulus, specifically the glomerular basement membrane (GBM) was altered, electron microscopy (EM) images of the glomeruli were taken, analyzed and the thickness of the GBM was measured. The GBM thickness of *elmo1* ^*−/−*^ and *elmo2* ^*−/−*^, respectively showed no significant change to the GBM thickness of *elmo1* ^*+/+*^ (Figures 5A,D) and *elmo2* ^*+/+*^ (Figures 5B,E) glomeruli. In contrast, the GBM thickness of *elmo3* ^*−/−*^ was increased compared to *elmo3* ^*+/+*^ (Figures 5C,F). In summary, the loss of Elmo1, Elmo2 or Elmo3, respectively, led to different changes in the glomerulus morphology, indicating a divergent impact of the three Elmo proteins on the development of the zebrafish kidney.

### Elmo1 Regulates Adult Blood Glucose Homeostasis in Zebrafish

**FIGURE 6:**
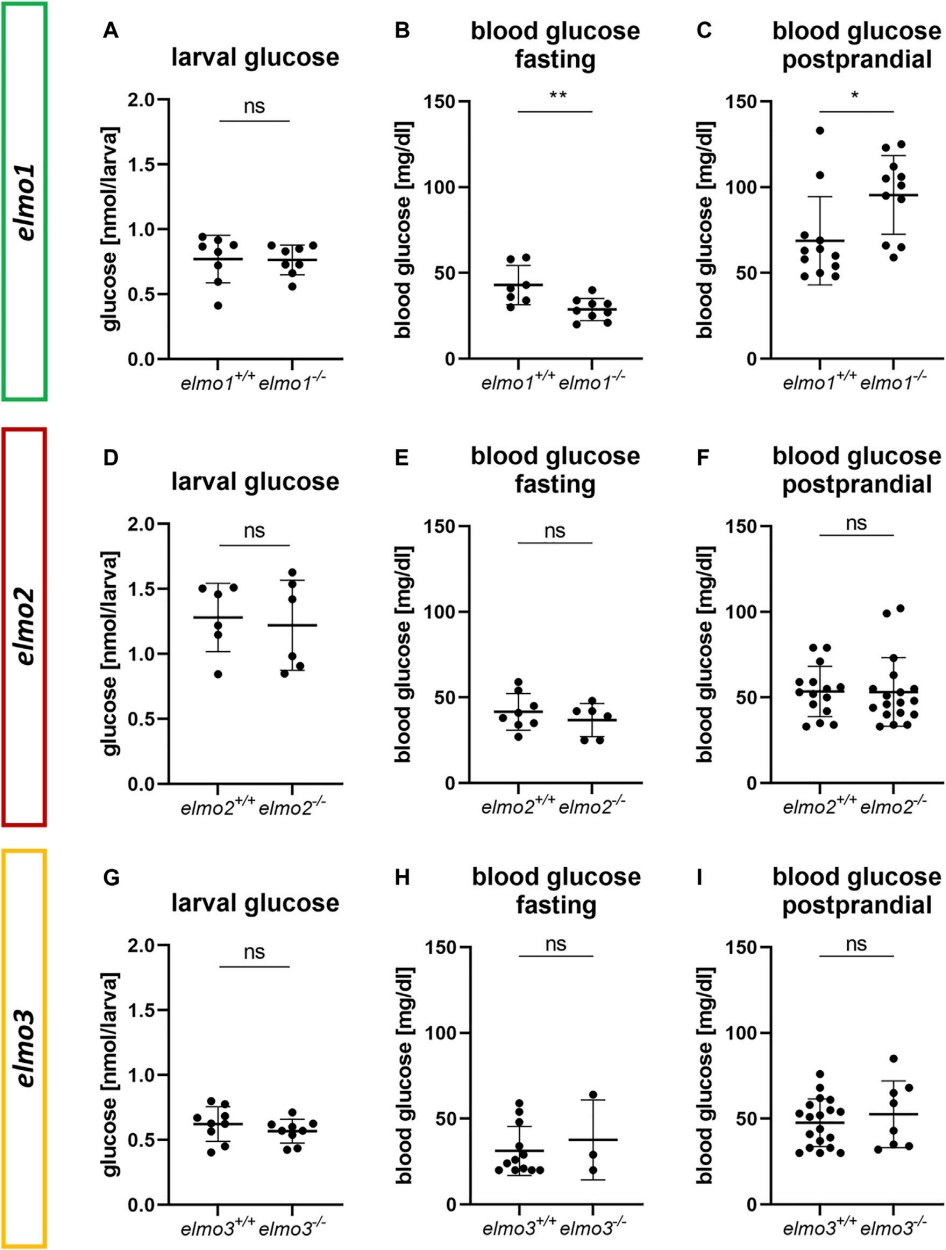
The loss of Elmo1 led to altered blood glucose levels in adult zebrafish. **(A)** Whole body glucose levels were not changed in *elmo1* ^*−/−*^ compared to *elmo1* ^*+/+*^ at 120 hpf. *n* = 8 samples per group, each containing 20 zebrafish larvae. **(B)** Fasting blood glucose levels were decreased in adult *elmo1* ^*−/−*^ *.* **(C)** Postprandial blood glucose levels were increased in adult *elmo1* ^*−/−*^. **(D)** Whole body glucose levels were not changed in *elmo2* ^*−/−*^ compared to *elmo2* ^*+/+*^ at 120 hpf. *n* = 6 samples per group, each containing 20 zebrafish larvae. **(E,F)** Fasting and postprandial blood glucose levels were unaltered in adult *elmo2* ^*−/−*^. **(G)** Whole body glucose levels were not changed in *elmo3* ^*−/−*^ compared to *elmo3* ^*+/+*^ at 120 hpf. *n* = 9 samples per group, each containing 20 zebrafish larvae. **(H,I)** Fasting and postprandial blood glucose levels were unaltered in adult *elmo3* ^*−/−*^. Blood glucose was obtained from animals of 7–15 mpf. Statistical analysis was done with *t*-test and Mann-Whitney test. *p < 0.05, **p < 0.01. ns, not significant; hpf, hours post fertilization; mpf, months post fertilization.

Previous studies have described the ELMO proteins as promotors for RAC1 activity (Gumienny et al., 2001; Brugnera et al., 2002; Katoh and Negishi, 2003; Cetinkaya et al., 2016; Tran et al., 2021) and RAC1 was shown to be an important regulator of the glucose homeostasis by ensuring the vesicular insulin transport by organizing the pancreatic cytoskeleton during glucose induced insulin secretion (Kowluru, 2011; Møller et al., 2019). Furthermore, a lack of RAC1 is associated with insulin resistance in humans and rodents (Sylow et al., 2013; Sylow et al., 2014; Raun et al., 2018) and ELMO2 and RAC1, both regulate the insulin dependent membrane translocation of the glucose transporter 4 (GLUT4) in muscle cells (Sun et al., 2016). To address the question if Elmo1, Elmo2 or Elmo3 maintain glucose homeostasis in zebrafish, whole body glucose content of larval (120 hpf) and blood glucose levels of adult (7–15 mpf) *elmo1* ^*−/−*^, *elmo2* ^*−/−*^, and *elmo3* ^*−/−*^ zebrafish were measured (Figure 6). Interestingly, adult blood glucose levels of fasted *elmo1* ^*−/−*^ zebrafish were decreased compared to *elmo1* ^*+/+*^ zebrafish (Figure 6B), whereas postprandial blood glucose levels were increased (Figure 6C). In *elmo2* ^*−/−*^ and *elmo3* ^*−/−*^ adult blood glucose levels, both fasting and postprandial, were unaltered compared to *elmo2* ^*+/+*^ (Figures 6E,F) and *elmo3* ^*+/+*^ (Figures 6H,I) and also the whole body glucose between *elmo1* ^*−/−*^, *elmo2* ^*−/−*^ and *elmo3* ^*−/−*^, and *elmo1* ^*+/+*^, *elmo2* ^*+/+*^ and *elmo3* ^*+/+*^ larvae at 120 hpf showed no differences (Figures 6A,D,G). In conclusion, these data have identified an important impact and thereby a novel function of Elmo1 in maintaining the regulation of blood glucose levels in adult zebrafish, thereby functionally differentiating itself from Elmo2 and Elmo3.

### Elmo Proteins Transcriptionally Modulate the Vascular and Neuronal Development of Zebrafish Larvae

To identify underlying pathways of the observed alterations in the vasculature, kidney and in glucose homeostasis in *elmo1* ^*−/−*^, *elmo2* ^*−/−*^, and *elmo3* ^*−/−*^ zebrafish and to reveal novel cellular mechanisms regulated by the Elmo proteins, we analyzed the regulation of the transcriptome with RNA sequencing data from zebrafish larvae at 120 hpf based on the Kyoto Encyclopedia of Genes and Genomes (KEGG) database for signaling pathways and based on the Gene Ontology Biological Process (GOBP) database for cell processes and mechanisms.

The KEGG based analysis revealed that out of 150 analyzed pathways, 11 were significantly regulated in *elmo1* ^*−/−*^, 9 in *elmo2* ^*−/−*^, and 62 in *elmo3* ^*−/−*^ mutants (Supplementary Table S1). These results suggested a strong impact of Elmo3 on the transcriptional regulation compared to the weaker regulation through the loss of Elmo1 or Elmo2. The altered transcriptional regulation of over one third of the analyzed pathways in *elmo3* ^*−/−*^ zebrafish might be a possible cause for its reduced viability. Interestingly, only three pathways in all three *elmo* mutants were found to be regulated in the same direction (Ribosome—upregulated; ECM-receptor interaction—downregulated; Focal adhesion—downregulated). Comparisons between two *elmo* mutants only, also exhibited solely four pathways regulated in the same direction between *elmo1* and *elmo2*, five between *elmo1* and *elmo3* and three between *elmo2* and *elmo3*, indicating only minor functional similarities in the regulation of the larval transcriptome Elmo1, Elmo2 and Elmo3. The downregulation of ECM-receptor interaction might affect organs depending on a correctly regulated ECM and could be a cause for structural changes as observed in the glomeruli of *elmo1* ^*−/−*^, *elmo2* ^*−/−*^, and *elmo3* ^*−/−*^ zebrafish.

**FIGURE 7:**
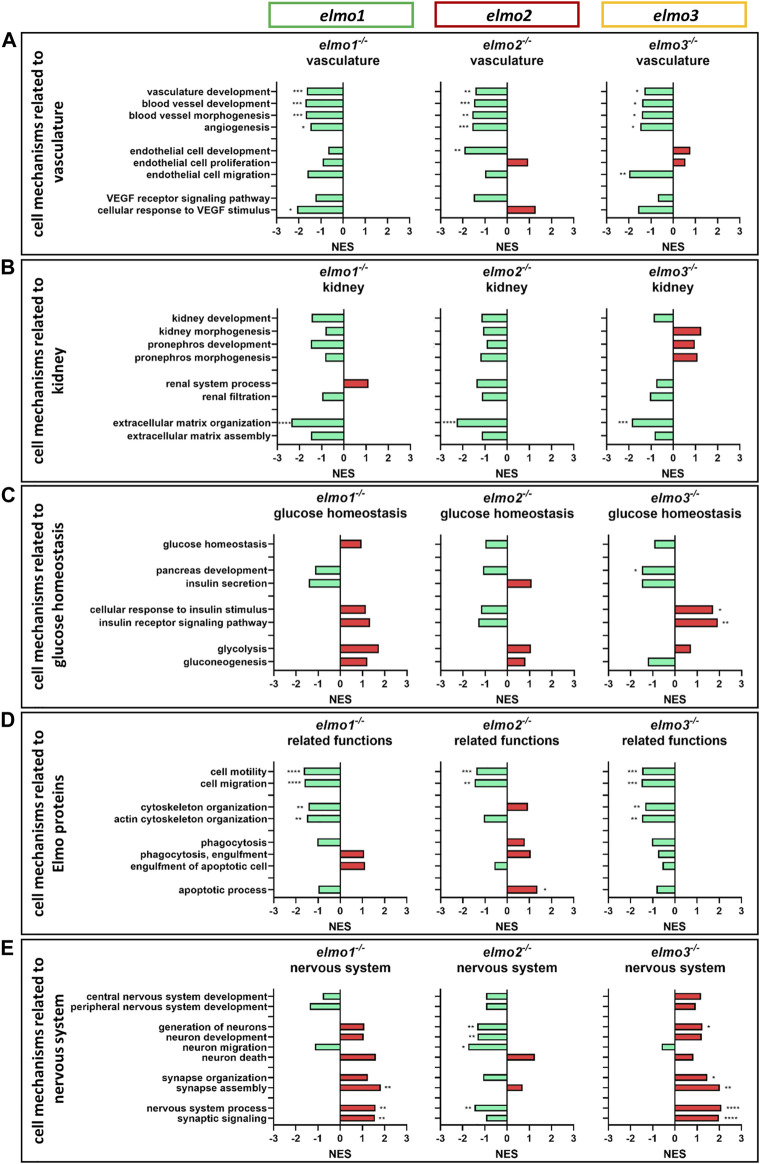
The loss of Elmo1, Elmo2 and Elmo3 led to similar and different changes in the regulation of the transcriptome in larval zebrafish. Comparison of the regulation of cell mechanisms in *elmo1* ^*−/−*^, *elmo2* ^*−/−*^ and *elmo3* ^*−/−*^ zebrafish larvae, respectively, compared to *elmo1* ^*+/+*^, *elmo2* ^*+/+*^ and *elmo3* ^*+/+*^. Gene Ontology Biological Process (GOBP) based analysis of cell mechanisms of the transcriptome using RNA-sequencing data from zebrafish larvae at 120 hpf. *n* = 6 samples per genotype, each containing 30 zebrafish larvae. Regulation of the gene sets is given as normalized enrichment score (NES). **(A)** Cell mechanisms related to vascular development were generally downregulated in *elmo1* ^*−/−*^, *elmo2* ^*−/−*^ and *elmo3* ^*−/−*^. **(B)** Cell mechanisms related to kidney development and function showed a downregulation of extracellular matrix organization in *elmo1* ^*−/−*^, *elmo2* ^*−/−*^ and *elmo3* ^*−/−*^. **(C)** Cell mechanisms related to glucose homeostasis were differently regulated in *elmo3* ^*−/−*^. **(D)** Cell mechanisms related to further functions of Elmo proteins showed a general downregulation in *elmo1* ^*−/−*^ and *elmo3* ^*−/−*^ and different changes in *elmo2* ^*−/−*^. **(E)** Cell mechanisms related to the nervous system were generally upregulated in *elmo1* ^*−/−*^ and *elmo3* ^*−/−*^ and downregulated in *elmo2* ^*−/−*^. hpf, hours post fertilization; NES, normalized enrichment score.

GOBP based analyses (Figures 7A–E) showed a downregulation of the vascular development, blood vessel development, blood vessel morphogenesis and angiogenesis mechanisms (Figure 7A) in the transcriptomes of *elmo1* ^*−/−*^, *elmo2* ^*−/−*^, and *elmo3* ^*−/−*^ zebrafish larvae, indicating that in early development the transcriptional regulation of the vasculature overlaps. These results correlated with the data of Elmo1 as a promoting factor of the trunk vasculature (Epting et al., 2010; Schäker et al., 2015) and the retinal vasculature (Figure 3) and suggested novel and so far unknown vascular functions of Elmo2 and Elmo3. However, mechanisms related to early kidney formation and function were not significantly regulated in any direction in *elmo1* ^*−/−*^, *elmo2* ^*−/−*^ and *elmo3* ^*−/−*^ zebrafish larvae (Figure 7B), albeit the data revealed an impact of the three Elmo proteins on the organization of the ECM, which is of major structural importance for the glomeruli and its GBM (Hobeika et al., 2017). These results supported the described function of ELMO1 regulating the expression of ECM components and their modulators (Shimazaki et al., 2005; Shimazaki et al., 2006) and the identified contribution of Elmo3 to the GBM (Figures 5C,F). The mechanisms related to glucose homeostasis were not significantly changed in larval *elmo1* ^*−/−*^ and *elmo2* ^*−/−*^ zebrafish (Figure 7C). Surprisingly, the *elmo3* ^*−/−*^ larval data exhibited a downregulated cellular response to insulin and pancreas development. Lastly, the transcriptome data revealed a transcriptional regulation of the three Elmo proteins on the nervous system process and neuronal development, which were upregulated in *elmo1* ^*−/−*^ and *elmo3* ^*−/−*^ (Figure 7E). This again correlated with known data of the ELMO proteins to regulate (Katoh and Negishi, 2003; Biersmith et al., 2011; Namekata et al., 2012; Lanoue et al., 2013; Makihara et al., 2018; Mikdache et al., 2019; Tran et al., 2021) and inhibit (Franke et al., 2012) neuronal development and revealed again a divergent function of Elmo1 and Elmo3 to Elmo2 in these processes.

In conclusion, these transcriptome analyses identified Elmo3 as an important transcriptional regulator in zebrafish larvae and highlight the three Elmo proteins as promotive factors for vascular development and regulators of the nervous system.

### The Loss of Elmo1 Led to an Increase of Amino Acids and the Loss of Elmo2 to an Increase of Fatty Acids in the Larval Metabolome

Because the transcriptome analysis exhibited several regulated pathways related to metabolic processes in *elmo3* ^*−/−*^ larvae (Supplementary Table S1), we subsequently assessed if the metabolome is affected through the loss of Elmo1, Elmo2 or Elmo3 by measuring different parts of the metabolome in *elmo1* ^*−/−*^, *elmo2* ^*−/−*^, and *elmo3* ^*−/−*^ larvae at 96 hpf (Supplementary Figures S7–S10). Surprisingly, the metabolome of *elmo3* ^*−/−*^ larvae just showed few changes (Supplementary Figures S7–S10) and did not well correlate with the transcriptional data. ADP and ATP were decreased in *elmo3* ^*−/−*^ compared to *elmo3* ^*+/+*^ (Supplementary Figure S9C) which was also the case in *elmo1* ^*−/−*^ compared to *elmo1* ^*+/+*^ larvae (Supplementary Figure S9A), suggesting a similar role in the regulation of the larval energy maintenance. However, *elmo1* ^*−/−*^ exhibited an increase of nine amino acids (Asp, Glu, Asn, Thr, Ala, Pro, Val, Ile, Leu) compared to *elmo1* ^*+/+*^ (Supplementary Figure S7A), whereas *elmo2* ^*−/−*^ (Supplementary Figure S7B) and *elmo3* ^*−/−*^ revealed hardly any changes (Supplementary Figure S7C). Measuring fatty acids showed an increase of eight fatty acids in *elmo2* ^*−/−*^ compared to *elmo2* ^*+/+*^ (Supplementary Figure S8B), while there were nearly no changes in *elmo1* ^*−/−*^ (Supplementary Figure S8A) and *elmo3* ^*−/−*^ (Supplementary Figure S8C). Both results indicate that Elmo1, Elmo2, and Elmo3 functionally diverge. Overall, the metabolome analyses of the *elmo1* ^*−/−*^, *elmo2* ^*−/−*^, and *elmo3* ^*−/−*^ larvae have not identified the Elmo proteins as major metabolic regulators.

## Discussion

**FIGURE 8:**
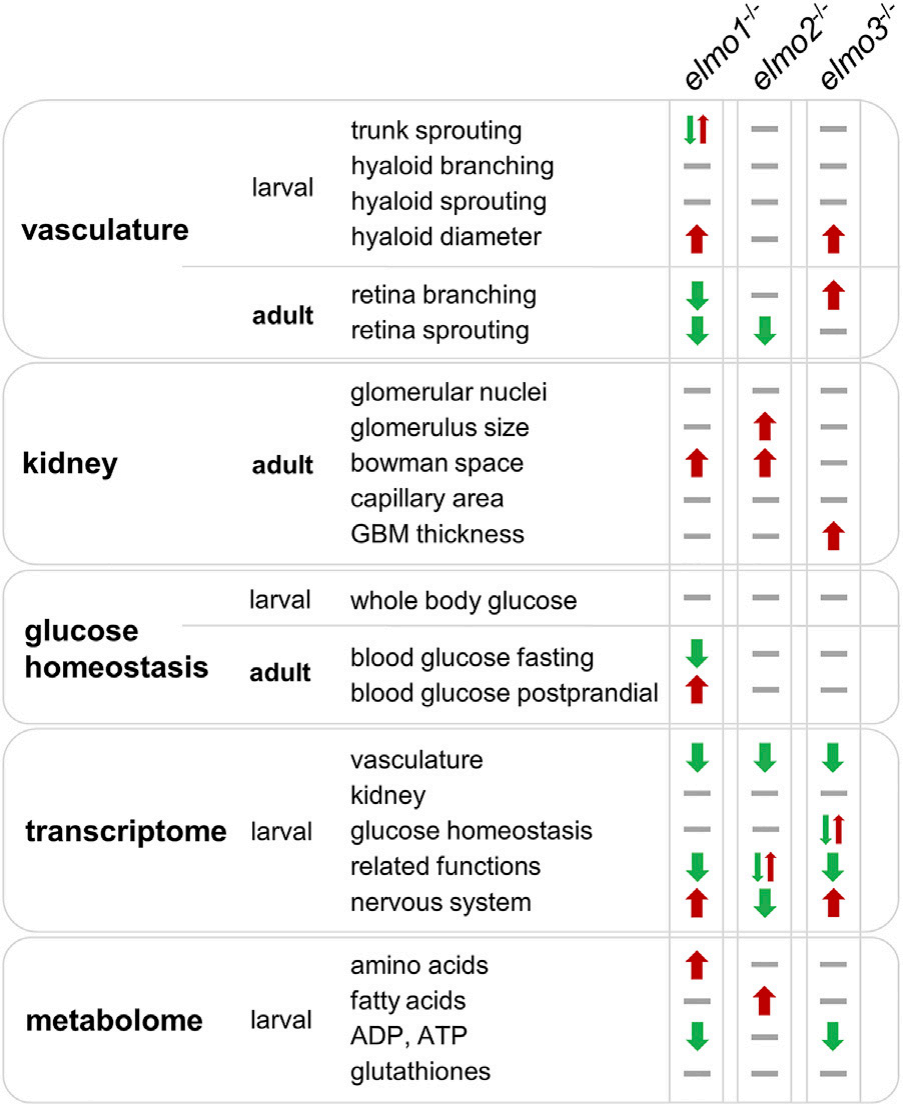
Comparison of *elmo1* ^*−/−*^, *elmo2* ^*−/−*^ and *elmo3* ^*−/−*^ phenotype in zebrafish indicates different functions of Elmo1, Elmo2 and Elmo3. Overview and comparison of the results of the phenotypical analysis of *elmo1* ^*−/−*^, *elmo2* ^*−/−*^ and *elmo3* ^*−/−*^ zebrafish in larval and adult stages indicate that Elmo1, Elmo2 and Elmo3 have not the same role in the regulation of the organism. Listed are all fields which were analyzed in this study and their outcome in the specific zebrafish line. Results are visualized with arrows and bars. Arrow up (red) indicates an increase, arrow down (green) indicates a decrease, one arrow up and one arrow down at once indicate different changes, bar (grey) indicates no change. GBM, glomerular basement membrane.

In this study, we established *elmo1*, *elmo2* and *elmo3* gene knockout zebrafish lines and performed comparative morphological, metabolic and transcriptome analyses. The data (Figure 8) showed that despite few similarities, *elmo1* ^*−/−*^, *elmo2* ^*−/−*^, and *elmo3* ^*−/−*^ zebrafish exhibited several different morphological changes in retina and kidney as well as alterations in the glucose homeostasis. These distinct findings suggest that the three Elmo proteins regulate similar and divergent biological processes and mechanisms and have a low functional redundancy.

In zebrafish, Elmo1 promotes early vascular development (Epting et al., 2010; Schäker et al., 2015). Our data strengthen this role of Elmo1 as a promotor of vascular formation by showing an impaired vascular sprouting additional to a decreased vascular formation in the retina of *elmo1* ^*−/−*^ zebrafish. An increased vessel thickness in larval hyaloids further suggests an impact of Elmo1 on vessel structure, expanding its regulatory capability in the vasculature. Interestingly, *elmo1* ^*−/−*^ zebrafish did not show vascular malformations in the larval trunk as described in a transient knockdown (Epting et al., 2010), which might be explained due to compensatory mechanisms occurring in permanent knockout models (El-Brolosy and Stainier, 2017; El-Brolosy et al., 2019). The promotive vascular function of Elmo1 was further supported through the larval transcriptome data showing the downregulation of vascular developmental processes. Interestingly, *elmo2* ^*−/−*^ and *elmo3* ^*−/−*^ zebrafish both exhibited a similar transcriptional regulation of the vasculature as seen in *elmo1* ^*−/−*^. In addition, individual changes in the vasculature were observed in two of the three knockout animals, as decreased vascular sprouting in adult retinae and an increased vessel diameter in larval hyaloids. Surprisingly, the adult retinae in *elmo3* ^*−/−*^ revealed an increased vascular formation, thereby showing an opposing change to *elmo1* ^*−/−*^. These findings indicate partially overlapping vascular functions of the three Elmo proteins; however, the varieties in the vascular phenotypes of *elmo1* ^*−/−*^, *elmo2* ^*−/−*^ and *elmo3* ^*−/−*^ also demonstrate differences in the regulation of vascular morphogenesis.

The second important observation of the study was the identification of an impact of Elmo1, Elmo2 and Elmo3 on the morphogenesis of glomerular structures. We observed changes in the form of an increased bowman space in *elmo1* ^*−/−*^, increased bowman space and glomerular hypertrophy in *elmo2* ^*−/−*^ and an increased GBM thickness in *elmo3* ^*−/−*^. These changes all are associated with pathologic kidney development. Glomerular hypertrophy can indicate hypertension (Wolf and Ziyadeh, 1999; Habib, 2018) and an increased GBM thickness is commonly associated with a reduced glomerular filtration rate (Thomson et al., 2008). Although the transcriptomes of *elmo1* ^*−/−*^, *elmo2* ^*−/−*^, and *elmo3* ^*−/−*^ zebrafish larvae exhibited unaltered processes associated with kidney development and function, the process of ECM organization and the ECM-receptor interaction pathway were strongly downregulated. These findings are now added to the described function of Elmo1 to regulate the ECM (Shimazaki et al., 2005; Shimazaki et al., 2006) and revealed novel insights into the involvement of Elmo2 and Elmo3 in ECM organization. Previous studies have shown that ELMO1 is linked to dysregulation of ECM, the primary component of the glomeruli and their GBM (Miner, 2012; Byron et al., 2014), resulting in structural changes in glomeruli and the GBM in particular (Shimazaki et al., 2006; Hathaway et al., 2016). Thus, Elmo2 and Elmo3 share a similar function with Elmo1 in regulating ECM proteins and all three proteins individually affect the glomerular morphogenesis and thereby the kidney health.

In addition the study identified Elmo1 as a novel glucose homeostasis regulator. Specifically, *elmo1* ^*−/−*^ zebrafish exhibited a decreased fasting and an increased postprandial blood glucose.

Consistent with the morphological findings in vasculature and kidney, our results of the transcriptome analysis showed parallels between *elmo1* ^*−/−*^, *elmo2* ^*−/−*^ and *elmo3* ^*−/−*^ zebrafish as well as several differences. Comparing the regulation of biological processes, multiple transcriptional patterns were shared between *elmo1* ^*−/−*^, *elmo2* ^*−/−*^ and *elmo3* ^*−/−*^ zebrafish larvae. Accordingly, cell motility, vascular development and extracellular matrix organization were equally downregulated. Furthermore, individual processes were similarly regulated only between *elmo1* ^*−/−*^ and *elmo3* ^*−/−*^, whereas *elmo2* ^*−/−*^ showed no or the opposite changes, as in cytoskeleton organization or nervous system process indicating functional similarities just between two of the three Elmo proteins. Additionally, this analysis revealed multiple distinct findings of the individual Elmo proteins, such as the increased apoptotic process in *elmo2* ^*−/−*^ or the increased response to insulin stimulus in *elmo3* ^*−/−*^, suggesting independent functions. Considering the total transcriptional regulation of signaling pathways, similarities appear only as exceptions as most pathways show different regulation patterns. In *elmo1* ^*−/−*^, *elmo2* ^*−/−*^, and *elmo3* ^*−/−*^ zebrafish, just three pathways showed a similar regulation, and only four pathways were regulated in parallel between *elmo1* ^*−/−*^ and *elmo2* ^*−/−*^, five between *elmo1* ^*−/−*^ and *elmo3* ^*−/−*^ and three between *elmo2* ^*−/−*^ and *elmo3* ^*−/−*^. Especially the dysregulation of one third of the analyzed pathways in the larval transcriptome of *elmo3* ^*−/−*^ zebrafish compared to *elmo3* ^*+/+*^ demonstrated a functional difference between the three Elmo proteins, as *elmo1* ^*−/−*^ and *elmo2* ^*−/−*^ showed a significantly smaller number of altered pathways. These overlapping findings of the morphologic and transcriptomic investigations are further supported by the metabolome analyses.

In conclusion, although all three Elmo proteins are guanine nucleotide exchange factors for Rac1, each individual Elmo protein possesses distinct functions, separating it from the other two. Therefore, they cannot be considered as functional homologues.

## Materials and Methods

### Study Approval

All experimental procedures on animals were approved by the local government authority, Regierungspräsidium Karlsruhe and by Medical Faculty Mannheim (license no: G-98/15 and I-19/02) and carried out in accordance with the approved guidelines.

### Zebrafish Husbandry

The zebrafish line *Tg(fli1:EGFP)* (Lawson and Weinstein, 2002) was used and raised as described (Kimmel et al., 1995) in normal husbandry environment. *Tg(fli1:EGFP)* was chosen because of its EGFP expressing endothelial cells. Embryos and larvae until 120 hpf were kept in egg water at 28.5°C with or without PTU (0.003%) to suppress pigmentation. Larvae older than 120 hpf and adult zebrafish were held in a 13-h light/11-h dark cycle. Zebrafish older than 90 days are referred to as adults. Adults were fed twice a day, fresh *Artemia Salina* in the morning and fish flake food at midday.

### Mutant Generation

To use the CRISPR/Cas9 system to generate mutations, guide RNA (gRNA) to target the zebrafish genes *elmo1* (exon 2), *elmo2* (exon 9) and *elmo3* (exon 2) were designed with ZiFiT Targeter Version 4.2 (primer are listed in Supplementary Table S2). It was cloned into a T7-driven promotor expression vector (pT7-gRNA; Addgene) for each gene, respectively. To generate mRNA, *in vitro* transcription was done. For the produced gRNA vector the T7 mMessage mMachine Kit (Invitrogen) was used and to get Cas9 mRNA the T3 MEGAshortscript Kit (Invitrogen) was used on a pT3TS-nCas9n vector (Addgene) following the protocol of the manufacturer. To generate the single knockout mutants, 1 nl KCl (0.1 M) solution containing gRNA (250 pg/nl) and Cas9 mRNA (200 pg/nl) was injected into the single cell stadium of an embryo (Jao et al., 2013). The injected F0 fish were analyzed for germline transmission and the mutated fish were crossed with *Tg(fli1:EGFP)* to generate heterozygous mutants. Genotyping was performed through Sanger sequencing or gel electrophoretic separation of PCR products (primer are listed in Supplementary Table S2). Sequencing results were analyzed with benchling (benchling.com) and PolyPeakParser (Hill et al., 2014).

### Preparation of Adult Zebrafish and Blood Glucose Measurement

For experiments with animals with a gene knockout, littermates were always used as control. Adult zebrafish were put into single boxes in the afternoon the day before preparation. Blood glucose measurement was either done after 21–23 h fasting or postprandial after feeding 1 h with 0.5 g flakes following 1 h in fresh water without food. Before blood glucose measurement and preparation fish were euthanized in an ice-cold water bath for 2 min. Blood was directly taken from the caudal vessel and measured with a glucometer (Freestyle Abbott) (Zang et al., 2015). Subsequently, fish were decapitated and transferred into ice-cold PBS for organ isolation. Organs were either snap frozen in liquid nitrogen for RNA isolation or put in 4% PFA/PBS for 24 h for visualization *via* confocal microscopy and periodic acid-Schiff (PAS) staining or in 3% glutaraldehyde in cacodylate (0.1 M, pH 7.4) for visualization *via* electron microscopy.

### Microscopy and Analysis of Vascular Structures in Larvae and Adults

Vascular structures of larval and adult zebrafish were imaged with the confocal fluorescence microscope (Leica DM6000 B) with a scanner (Leica TCS SP5 DS) using a 20 × 0.7 objective. Images were analyzed with Leica Application Suite X and ImageJ.

For analysis of the larval trunk vasculature, larvae at 32 and 96 hpf were anaesthetized in 0.003% tricaine and observed with a Leica MSV269. The first six intersegmental vessels (ISVs) were skipped. The next 17 ISVs on both sides of the larva were observed for deformations and additional sprouts. Imaging was done as described above.

For analysis of the hyaloid vasculature, larvae at 120 hpf were anaesthetized in 0.003% tricaine and fixated in 4% PFA/PBS for 24 h. Afterwards fixated larvae were incubated in 0.25% Trypsin/EDTA solution buffered with TRIS HCl (1.5 M, pH 7.8) for 80 min at room temperature during gentle movement on a shaker. Subsequently, larvae were washed three times for 10 min with PBS and stored in PBS at 4°C until preparation. Hyaloids were isolated on an agarose plate and placed and on an object slide in a drop of water. Imaging was done as described above.

Imaging the adult retina vasculature was done as described (Wiggenhauser et al., 2017). For preparation, the fixated eye was transferred into cold PBS on an agarose plate for dissection. The retina was isolated and put on PBS on an object slide, cut and covered in mounting medium with a cover slip. Imaging was done as described above.

### Analysis of Kidney Morphology

For analysis of the glomerular structure, kidneys were fixated in 4% PFA/PBS in preparation for PAS staining. Fixated kidneys were embedded in paraffin and cut in 4 µm thick sections with a Leica RM2235 microtome and placed on an object slide. After deparaffinization, sections were put in 1% periodic acid for 10 min, washed in distilled water and put Schiff’s reagent for 20 min, followed by three times for 2 min in SO_2_ water. Afterwards sections were rinsed in running tap water, stained in hematoxylin solution and rinsed again, each for 5 min. Stained sections were dehydrated with ethanol solutions and mounted with mounting medium. Brightfield imaging was done with a scanner (Zeiss Axio Scan.Z1). Analysis of glomerular structure was done as described previously (Wiggenhauser et al., 2022). In brief, just sections were used at the point or near the point where the arterioles go into or out of the glomerulus to thereby try to analyze the glomerulus at its most concentric point as possible to obtain maximal comparability. For analysis the software ImageJ was used. Area size measurements were done with the Measure and with the Threshold tool. Nuclei numbers were counted manually.

For analysis of the glomerular basement membrane, kidneys were fixated in 3% glutaraldehyde in cacodylate (0.1 M, pH 7.4), cut in 1 mm^3^ pieces and postfixed in 1% aqueous osmium tetroxide for 1 h at 4°C. Subsequently, they were rinsed with water, dehydrated using ethanol solutions, transferred into propylene oxide and embedded in Epoxy resin (glycidether 100). With an ultramicrotome (Reichert Ultracut E) semithin (1 µm) and ultrathin sections (60–80 nm) were cut. To identify the glomeruli, the semithin sections were stained with methylene blue and analyzed under a light microscope (Olympus) at ×200 magnification. Ultrathin sections were treated with uranyl acetate and lead citrate and examined with a transmission electron microscope (JEM 1400), equipped with a 2k TVIPS CCD camera (TemCam F216) at ×3,000–×10,000 magnification. Analysis was done with EMMeasure.

### Collection of Zebrafish Larval Samples

If not described differently, zebrafish larvae were anaesthetized in 0.003% tricaine, collected and snap frozen in liquid nitrogen. Homogenization was achieved with a 1 ml syringe and a 25 G needle.

### Whole Body Glucose Measurement in Larvae

Twenty larvae at 120 hpf were collected per sample. Samples were homogenized in assay buffer. A glucose assay (MAK263, Sigma-Aldrich) was used to determine the glucose levels following the protocol of the manufacturer. Fluorometric detection was achieved with a plate reader (Tecan Infinite M200).

### Reverse-Transcription Polymerase Chain Reaction

Thirty larvae were collected per sample. Samples were homogenized in 1% 2-mercaptoethanol in RLT buffer. Total RNA was isolated with the RNeasy Mini Kit (Qiagen) following the protocol of the manufacturer. cDNA was synthesized from 1 µg RNA with the Maxima First Strand cDNA Synthesis Kit (Thermo Fisher Scientific) according to the protocol of the manufacturer. Standard PCRs were applied with the generated cDNA. Mutations (deletion or insertion) were made visible through gel electrophoretic separation of PCR products (primer are listed in Supplementary Table S2).

### Reverse-Transcription Quantitative Polymerase Chain Reaction

Thirty larvae or one total organ was collected per sample. Samples were homogenized in 1% 2-mercaptoethanol in RLT buffer. Total RNA was isolated with the RNeasy Mini Kit (Qiagen) following the protocol of the manufacturer. cDNA was synthesized from 1 µg RNA with the Maxima First Strand cDNA Synthesis Kit (Thermo Fisher Scientific) according to the protocol of the manufacturer. For RT-qPCR Power SYBR™ Green PCR Master Mix Kit (Thermo Fisher Scientific) and 96 well reaction plates were used with a QuantStudio™ 3 Real-Time PCR System (Thermo Fisher Scientific) (primer are listed in Supplementary Table S2).

### RNA-Sequencing and Transcriptome Analysis

Thirty larvae at 120 hpf were collected per sample. Samples were homogenized in 1% 2-mercaptoethanol in RLT buffer. Total RNA was isolated with the RNeasy Mini Kit (Qiagen) following the protocol of the manufacturer. Library construction and sequencing were performed by the Beijing Genomic Institution (BGI) with a BGISEQ-500. Sequencing analysis was performed by the Next-Generation Sequencing (NGS) Core Facility of the medical faculty Mannheim as described (Lou et al., 2020). Raw RNA sequencing data are available at GEO (Gene Expression Omnibus, NCBI) under the accession number: GSE197827.

### Metabolome Analysis

For sample collection, zebrafish larvae at 96 hpf were anaesthetized in 0.003% tricaine, collected and snap frozen in liquid nitrogen. Forty larvae were collected per sample. Measurements of amino acids, fatty acids, adenosine compounds and glutathione were performed by the Metabolomics Core Technology Platform from the Centre of Organismal Studies Heidelberg as described before (Lodd et al., 2019).

### Protein Sequence Alignment

Amino acid sequence alignment was achieved with the UniProt Align tool (https://www.uniprot.org/align/) with zebrafish Elmo1 (UniProt ID: Q6NV39), Elmo2 (UniProt ID: A0A0G2KRJ3) and Elmo3 (UniProt ID: F1QSV8) and human ELMO1 (UniProt ID: Q92556), ELMO2 (UniProt ID: Q96JJ3) and ELMO3 (UniProt ID: Q96BJ8).

### Statistics

Data are given as mean with standard deviation. For the comparison of two groups of data, datasets were tested for Gaussian distribution. Then statistical significance between those groups was analyzed with the *t*-test (if data was normally distributed) or with the Mann-Whitney test (if data was not normally distributed). To determine statistical significance between the genotype distribution of zebrafish lines and the mendelian inheritance, the chi square test was applied. To determine statistical significance between the survival of zebrafish lines, a logrank test was applied. *p*-values were given as: *p < 0.05, **p < 0.01, ***p < 0.001, ****p < 0.0001. GraphPad Prism 9.3.1 was used for statistical analyses.

## Data Availability

The datasets presented in this study can be found in online repositories. The name of the repository and accession number can be found below: NCBI Gene Expression Omnibus; accession number GSE197827.
