# Supplementary material for: Comparative Morphological, Metabolic and Transcriptome Analyses in elmo1 −/− , elmo2 −/− , and elmo3 −/− Zebrafish Mutants Identified a Functional Non-Redundancy of the Elmo Proteins
Source: Front Cell Dev Biol. 2022 Jul 8;10:918529. doi: 10.3389/fcell.2022.918529 (PMC9304559; doi:10.3389/fcell.2022.918529)
Supplement: Supplementary file 1 [file Presentation1.pptx]

## Slide 1
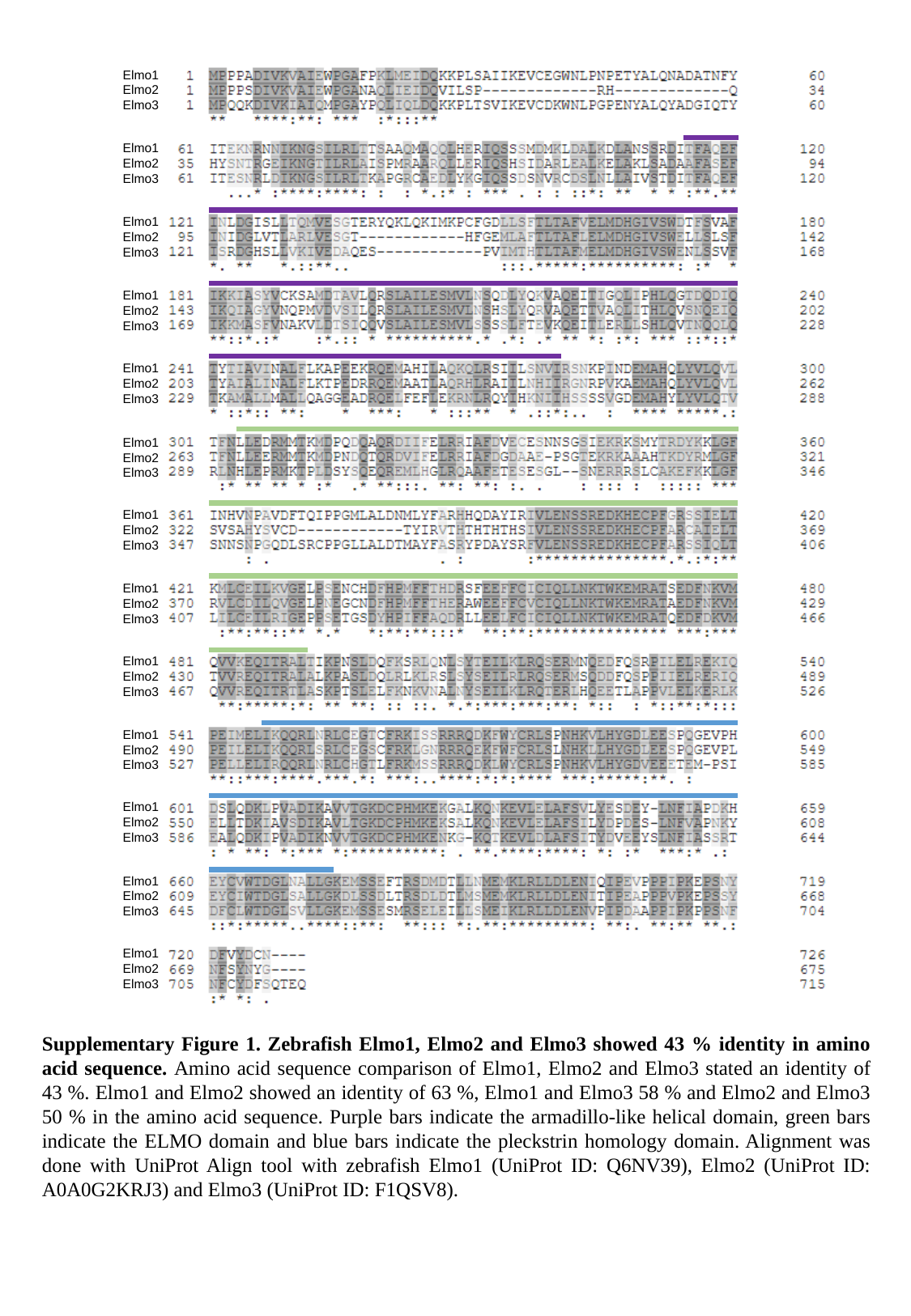

Elmo1
Elmo2
Elmo3
Elmo1
Elmo2
Elmo3
Elmo1
Elmo2
Elmo3
Elmo1
Elmo2
Elmo3
Elmo1
Elmo2
Elmo3
Elmo1
Elmo2
Elmo3
Elmo1
Elmo2
Elmo3
Elmo1
Elmo2
Elmo3
Elmo1
Elmo2
Elmo3
Elmo1
Elmo2
Elmo3
Elmo1
Elmo2
Elmo3
Elmo1
Elmo2
Elmo3
Elmo1
Elmo2
Elmo3
Supplementary Figure 1. Zebrafish Elmo1, Elmo2 and Elmo3 showed 43 % identity in amino acid sequence. Amino acid sequence comparison of Elmo1, Elmo2 and Elmo3 stated an identity of 43 %. Elmo1 and Elmo2 showed an identity of 63 %, Elmo1 and Elmo3 58 % and Elmo2 and Elmo3 50 % in the amino acid sequence. Purple bars indicate the armadillo-like helical domain, green bars indicate the ELMO domain and blue bars indicate the pleckstrin homology domain. Alignment was done with UniProt Align tool with zebrafish Elmo1 (UniProt ID: Q6NV39), Elmo2 (UniProt ID: A0A0G2KRJ3) and Elmo3 (UniProt ID: F1QSV8).

## Slide 2
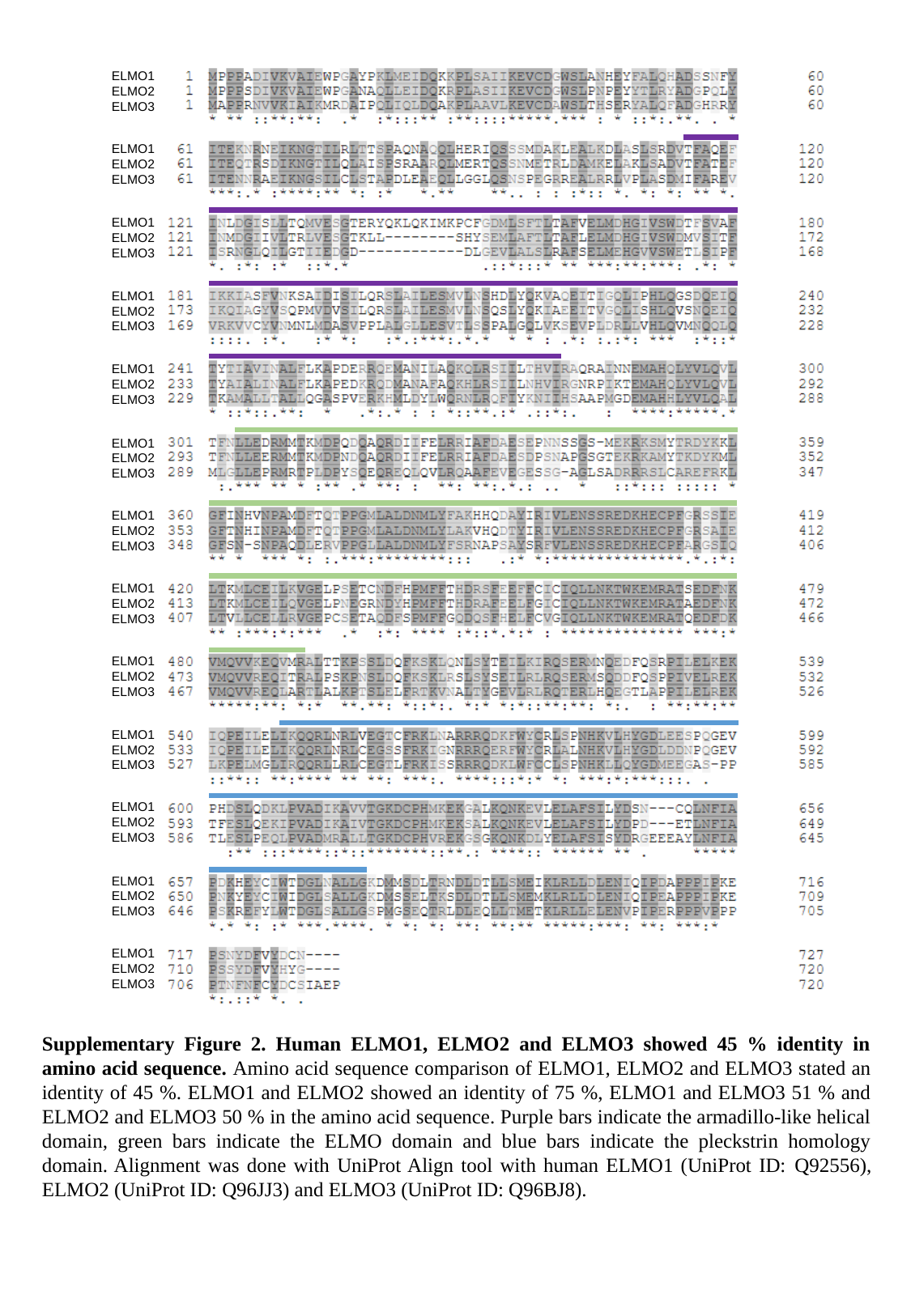

ELMO1
ELMO2
ELMO3
ELMO1
ELMO2
ELMO3
ELMO1
ELMO2
ELMO3
ELMO1
ELMO2
ELMO3
ELMO1
ELMO2
ELMO3
ELMO1
ELMO2
ELMO3
ELMO1
ELMO2
ELMO3
ELMO1
ELMO2
ELMO3
ELMO1
ELMO2
ELMO3
ELMO1
ELMO2
ELMO3
ELMO1
ELMO2
ELMO3
ELMO1
ELMO2
ELMO3
ELMO1
ELMO2
ELMO3
Supplementary Figure 2. Human ELMO1, ELMO2 and ELMO3 showed 45 % identity in amino acid sequence. Amino acid sequence comparison of ELMO1, ELMO2 and ELMO3 stated an identity of 45 %. ELMO1 and ELMO2 showed an identity of 75 %, ELMO1 and ELMO3 51 % and ELMO2 and ELMO3 50 % in the amino acid sequence. Purple bars indicate the armadillo-like helical domain, green bars indicate the ELMO domain and blue bars indicate the pleckstrin homology domain. Alignment was done with UniProt Align tool with human ELMO1 (UniProt ID: Q92556), ELMO2 (UniProt ID: Q96JJ3) and ELMO3 (UniProt ID: Q96BJ8).

## Slide 3
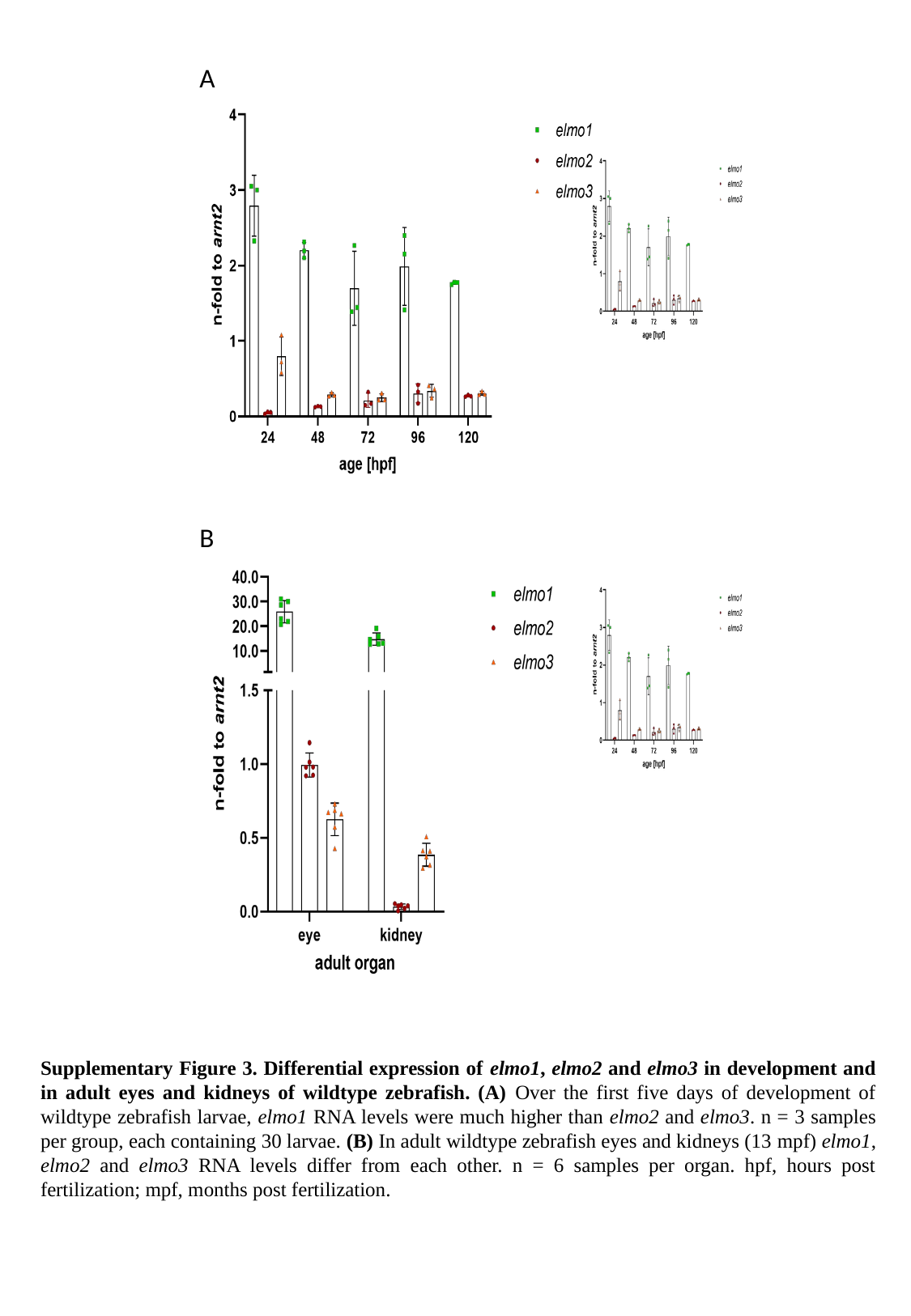

A
B
Supplementary Figure 3. Differential expression of elmo1, elmo2 and elmo3 in development and in adult eyes and kidneys of wildtype zebrafish. (A) Over the first five days of development of wildtype zebrafish larvae, elmo1 RNA levels were much higher than elmo2 and elmo3. n = 3 samples per group, each containing 30 larvae. (B) In adult wildtype zebrafish eyes and kidneys (13 mpf) elmo1, elmo2 and elmo3 RNA levels differ from each other. n = 6 samples per organ. hpf, hours post fertilization; mpf, months post fertilization.

## Slide 4
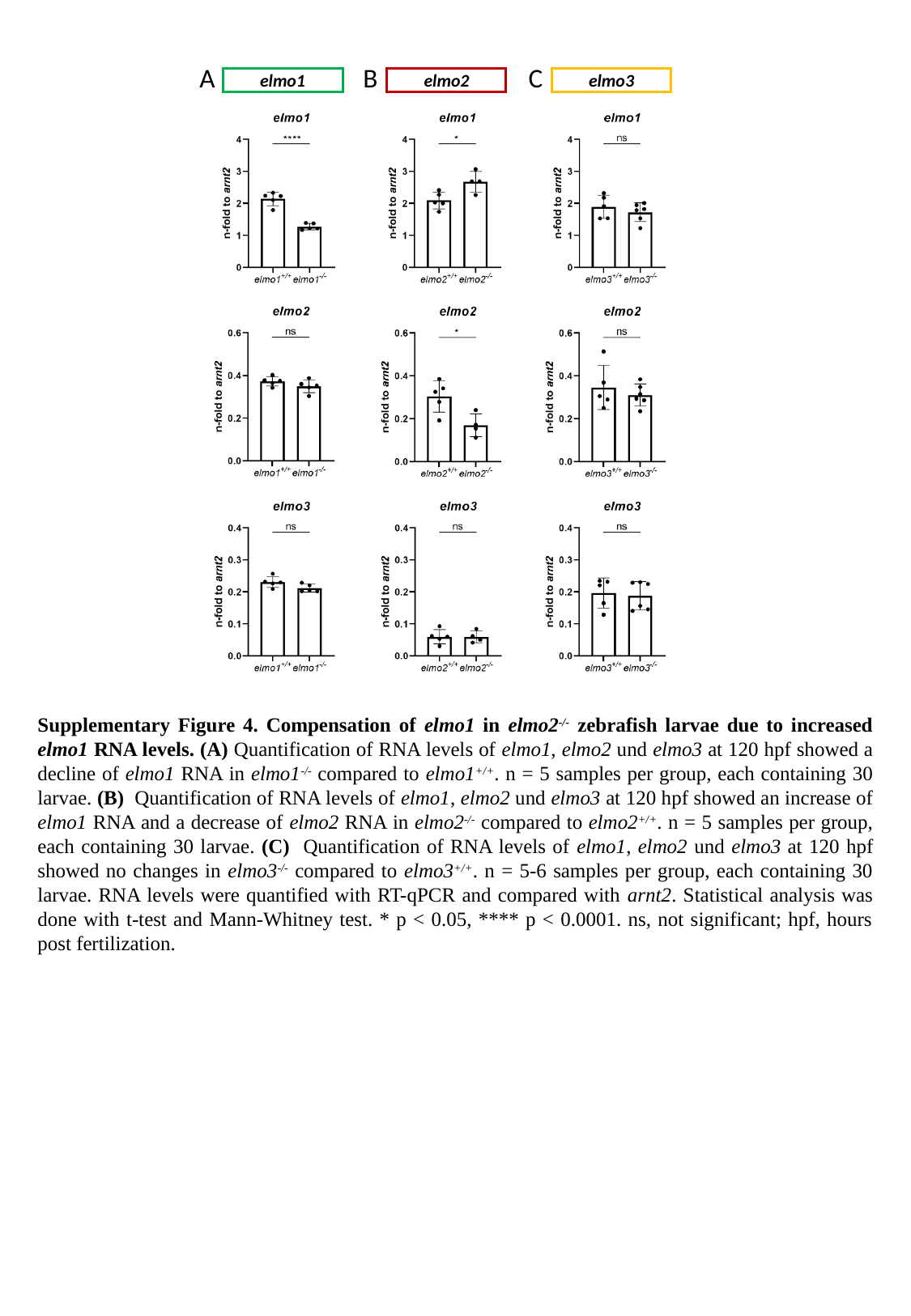

A
B
C
elmo2
elmo3
elmo1
Supplementary Figure 4. Compensation of elmo1 in elmo2-/- zebrafish larvae due to increased elmo1 RNA levels. (A) Quantification of RNA levels of elmo1, elmo2 und elmo3 at 120 hpf showed a decline of elmo1 RNA in elmo1-/- compared to elmo1+/+. n = 5 samples per group, each containing 30 larvae. (B) Quantification of RNA levels of elmo1, elmo2 und elmo3 at 120 hpf showed an increase of elmo1 RNA and a decrease of elmo2 RNA in elmo2-/- compared to elmo2+/+. n = 5 samples per group, each containing 30 larvae. (C) Quantification of RNA levels of elmo1, elmo2 und elmo3 at 120 hpf showed no changes in elmo3-/- compared to elmo3+/+. n = 5-6 samples per group, each containing 30 larvae. RNA levels were quantified with RT-qPCR and compared with arnt2. Statistical analysis was done with t-test and Mann-Whitney test. * p < 0.05, **** p < 0.0001. ns, not significant; hpf, hours post fertilization.

## Slide 5
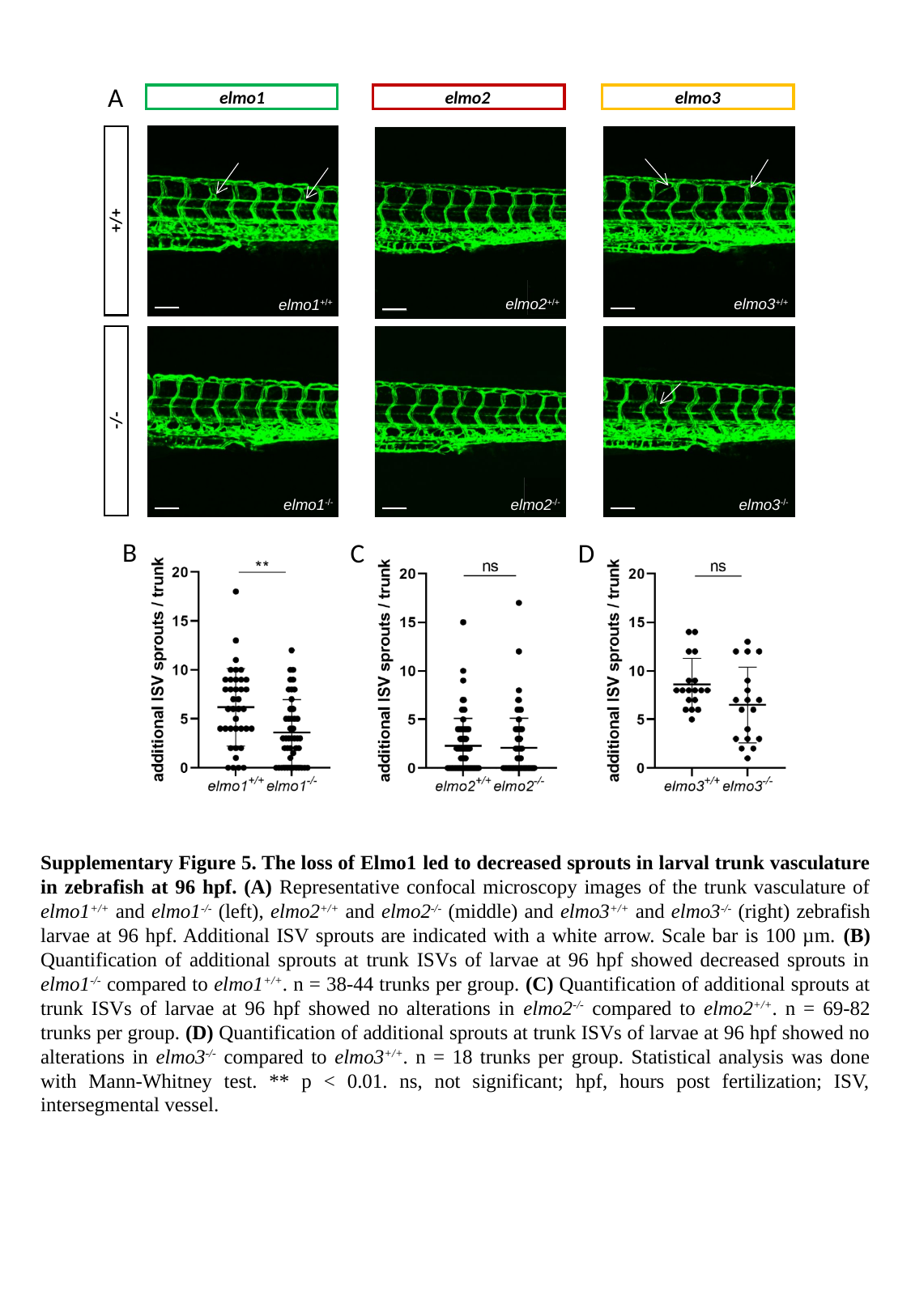

A
elmo1
elmo2
elmo3
+/+
elmo2+/+
elmo3+/+
elmo1+/+
-/-
elmo2-/-
elmo3-/-
elmo1-/-
B
C
D
Supplementary Figure 5. The loss of Elmo1 led to decreased sprouts in larval trunk vasculature in zebrafish at 96 hpf. (A) Representative confocal microscopy images of the trunk vasculature of elmo1+/+ and elmo1-/- (left), elmo2+/+ and elmo2-/- (middle) and elmo3+/+ and elmo3-/- (right) zebrafish larvae at 96 hpf. Additional ISV sprouts are indicated with a white arrow. Scale bar is 100 µm. (B) Quantification of additional sprouts at trunk ISVs of larvae at 96 hpf showed decreased sprouts in elmo1-/- compared to elmo1+/+. n = 38-44 trunks per group. (C) Quantification of additional sprouts at trunk ISVs of larvae at 96 hpf showed no alterations in elmo2-/- compared to elmo2+/+. n = 69-82 trunks per group. (D) Quantification of additional sprouts at trunk ISVs of larvae at 96 hpf showed no alterations in elmo3-/- compared to elmo3+/+. n = 18 trunks per group. Statistical analysis was done with Mann-Whitney test. ** p < 0.01. ns, not significant; hpf, hours post fertilization; ISV, intersegmental vessel.

## Slide 6
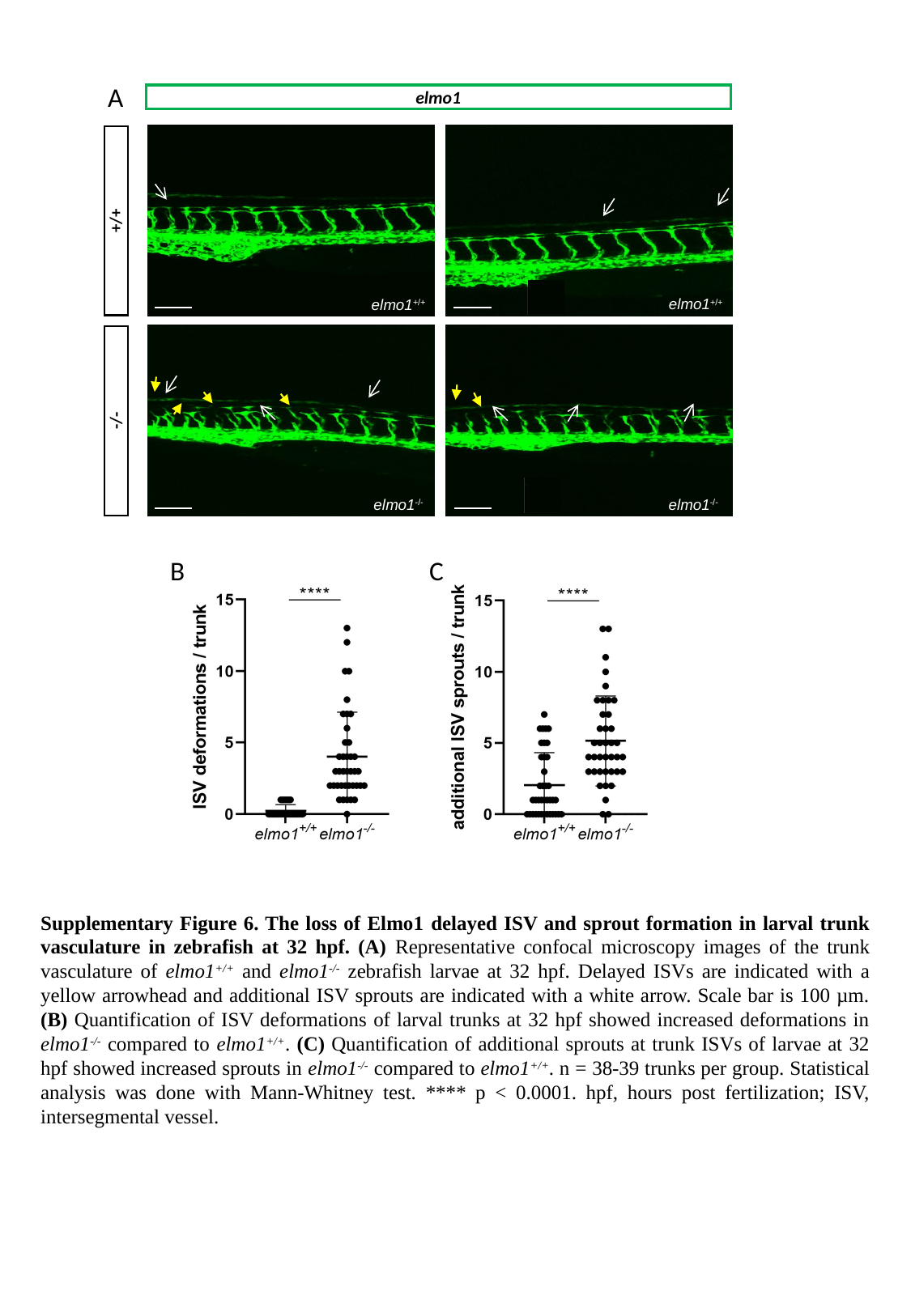

A
elmo1
+/+
elmo1+/+
elmo1+/+
-/-
elmo1-/-
elmo1-/-
B
C
Supplementary Figure 6. The loss of Elmo1 delayed ISV and sprout formation in larval trunk vasculature in zebrafish at 32 hpf. (A) Representative confocal microscopy images of the trunk vasculature of elmo1+/+ and elmo1-/- zebrafish larvae at 32 hpf. Delayed ISVs are indicated with a yellow arrowhead and additional ISV sprouts are indicated with a white arrow. Scale bar is 100 µm. (B) Quantification of ISV deformations of larval trunks at 32 hpf showed increased deformations in elmo1-/- compared to elmo1+/+. (C) Quantification of additional sprouts at trunk ISVs of larvae at 32 hpf showed increased sprouts in elmo1-/- compared to elmo1+/+. n = 38-39 trunks per group. Statistical analysis was done with Mann-Whitney test. **** p < 0.0001. hpf, hours post fertilization; ISV, intersegmental vessel.

## Slide 7
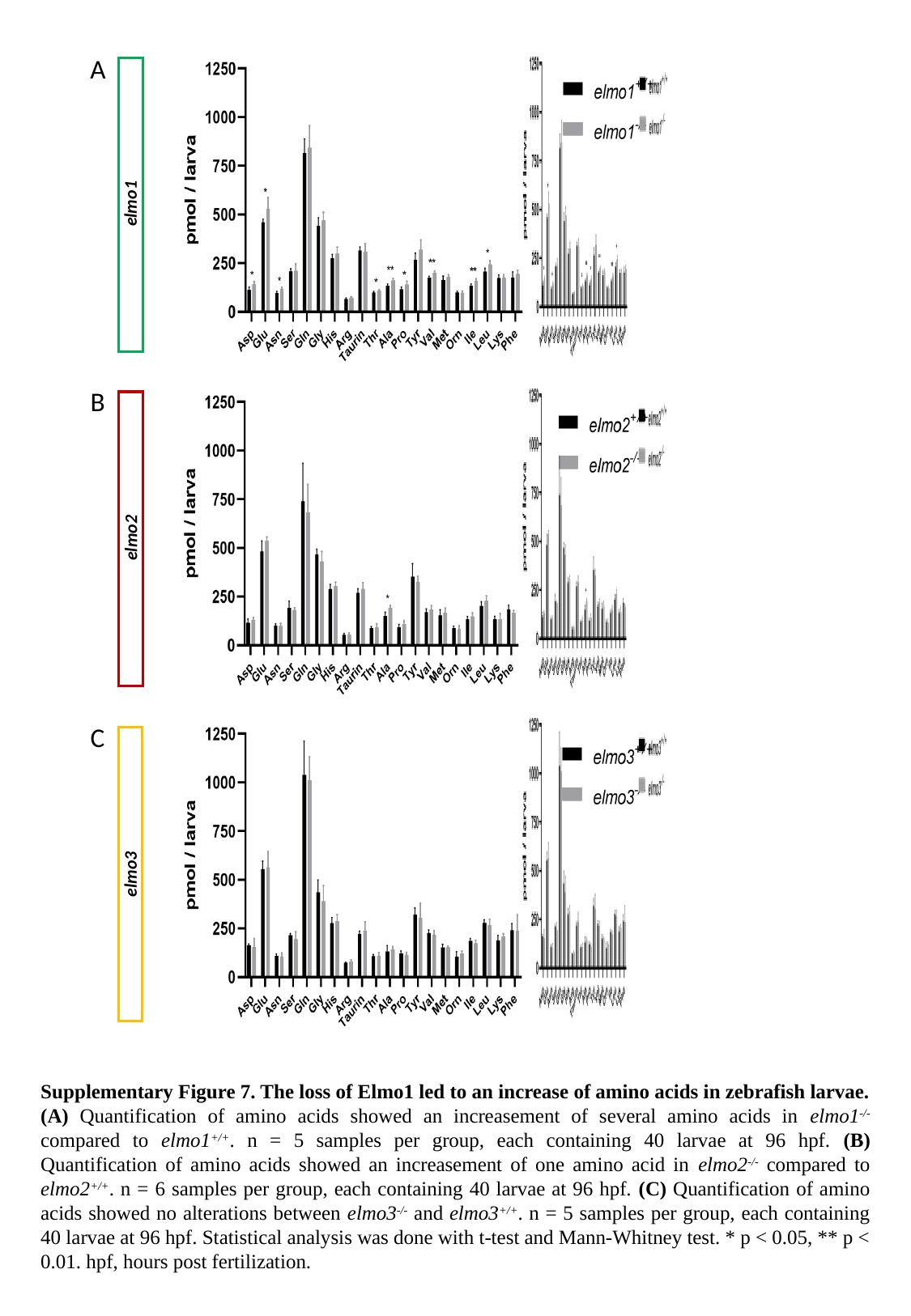

A
elmo1
B
elmo2
C
elmo3
Supplementary Figure 7. The loss of Elmo1 led to an increase of amino acids in zebrafish larvae. (A) Quantification of amino acids showed an increasement of several amino acids in elmo1-/- compared to elmo1+/+. n = 5 samples per group, each containing 40 larvae at 96 hpf. (B) Quantification of amino acids showed an increasement of one amino acid in elmo2-/- compared to elmo2+/+. n = 6 samples per group, each containing 40 larvae at 96 hpf. (C) Quantification of amino acids showed no alterations between elmo3-/- and elmo3+/+. n = 5 samples per group, each containing 40 larvae at 96 hpf. Statistical analysis was done with t-test and Mann-Whitney test. * p < 0.05, ** p < 0.01. hpf, hours post fertilization.

## Slide 8
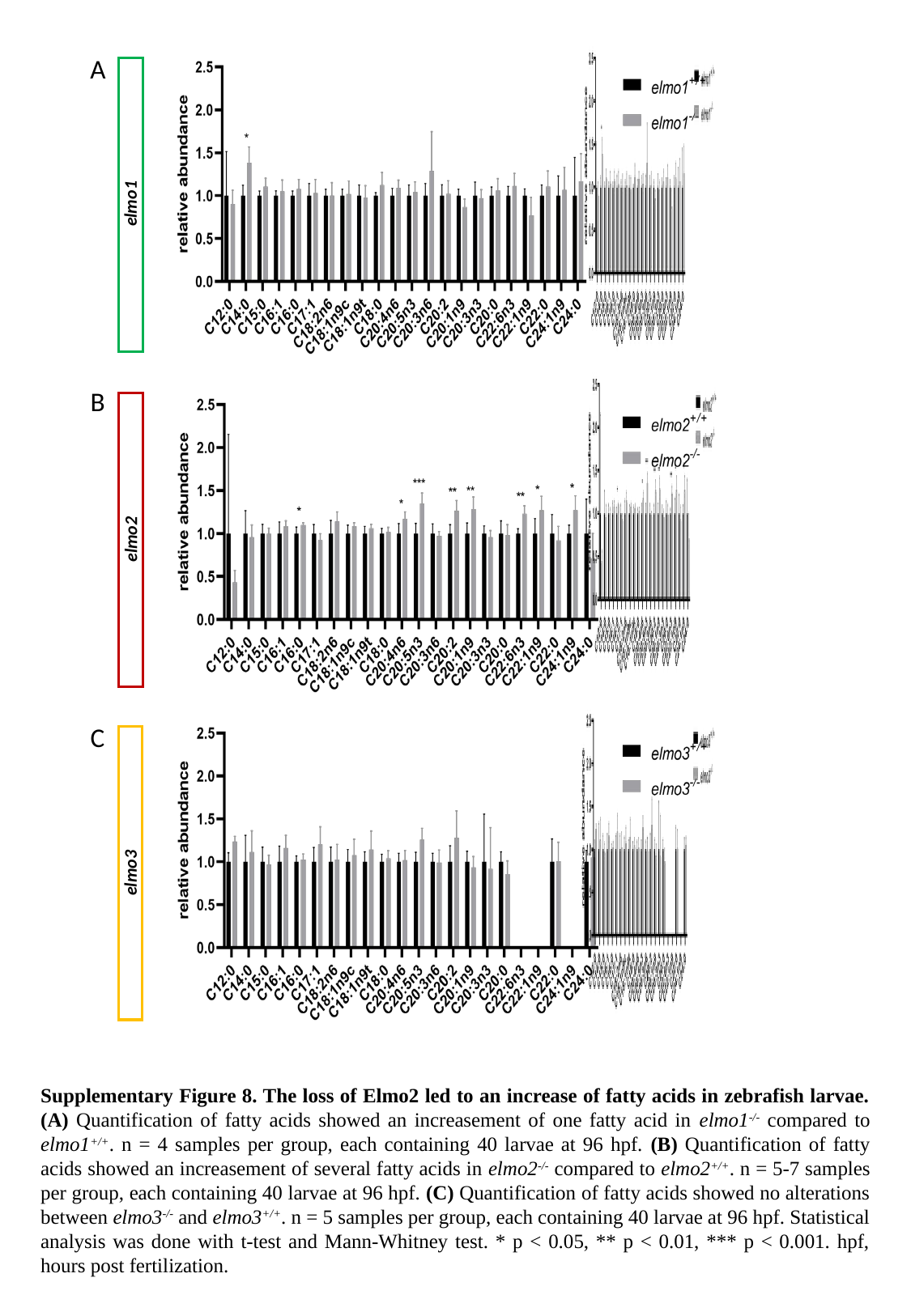

A
elmo1
B
elmo2
C
elmo3
Supplementary Figure 8. The loss of Elmo2 led to an increase of fatty acids in zebrafish larvae. (A) Quantification of fatty acids showed an increasement of one fatty acid in elmo1-/- compared to elmo1+/+. n = 4 samples per group, each containing 40 larvae at 96 hpf. (B) Quantification of fatty acids showed an increasement of several fatty acids in elmo2-/- compared to elmo2+/+. n = 5-7 samples per group, each containing 40 larvae at 96 hpf. (C) Quantification of fatty acids showed no alterations between elmo3-/- and elmo3+/+. n = 5 samples per group, each containing 40 larvae at 96 hpf. Statistical analysis was done with t-test and Mann-Whitney test. * p < 0.05, ** p < 0.01, *** p < 0.001. hpf, hours post fertilization.

## Slide 9
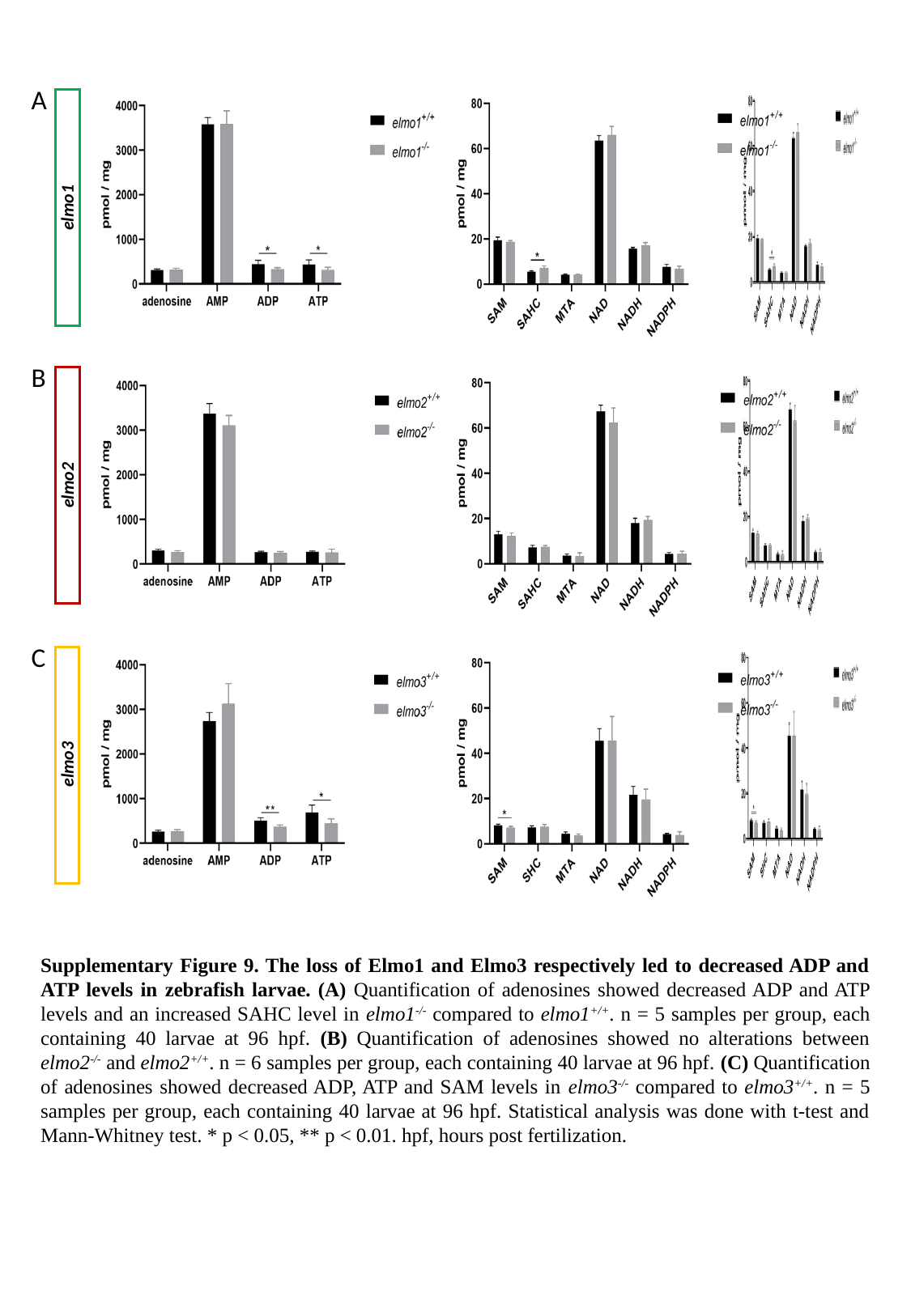

A
elmo1
B
elmo2
C
elmo3
Supplementary Figure 9. The loss of Elmo1 and Elmo3 respectively led to decreased ADP and ATP levels in zebrafish larvae. (A) Quantification of adenosines showed decreased ADP and ATP levels and an increased SAHC level in elmo1-/- compared to elmo1+/+. n = 5 samples per group, each containing 40 larvae at 96 hpf. (B) Quantification of adenosines showed no alterations between elmo2-/- and elmo2+/+. n = 6 samples per group, each containing 40 larvae at 96 hpf. (C) Quantification of adenosines showed decreased ADP, ATP and SAM levels in elmo3-/- compared to elmo3+/+. n = 5 samples per group, each containing 40 larvae at 96 hpf. Statistical analysis was done with t-test and Mann-Whitney test. * p < 0.05, ** p < 0.01. hpf, hours post fertilization.

## Slide 10
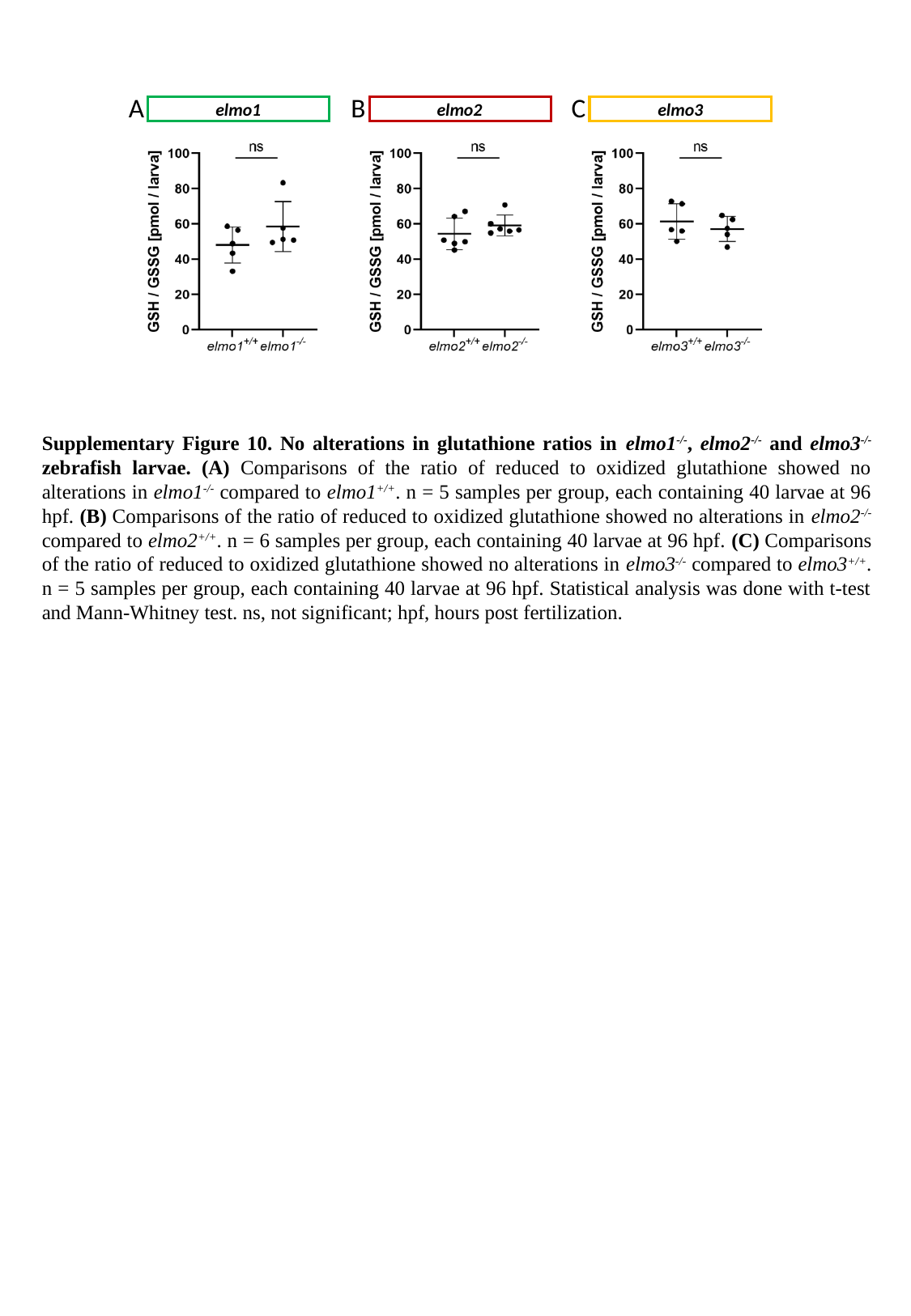

B
A
C
elmo1
elmo2
elmo3
Supplementary Figure 10. No alterations in glutathione ratios in elmo1-/-, elmo2-/- and elmo3-/- zebrafish larvae. (A) Comparisons of the ratio of reduced to oxidized glutathione showed no alterations in elmo1-/- compared to elmo1+/+. n = 5 samples per group, each containing 40 larvae at 96 hpf. (B) Comparisons of the ratio of reduced to oxidized glutathione showed no alterations in elmo2-/- compared to elmo2+/+. n = 6 samples per group, each containing 40 larvae at 96 hpf. (C) Comparisons of the ratio of reduced to oxidized glutathione showed no alterations in elmo3-/- compared to elmo3+/+. n = 5 samples per group, each containing 40 larvae at 96 hpf. Statistical analysis was done with t-test and Mann-Whitney test. ns, not significant; hpf, hours post fertilization.

## Slide 11
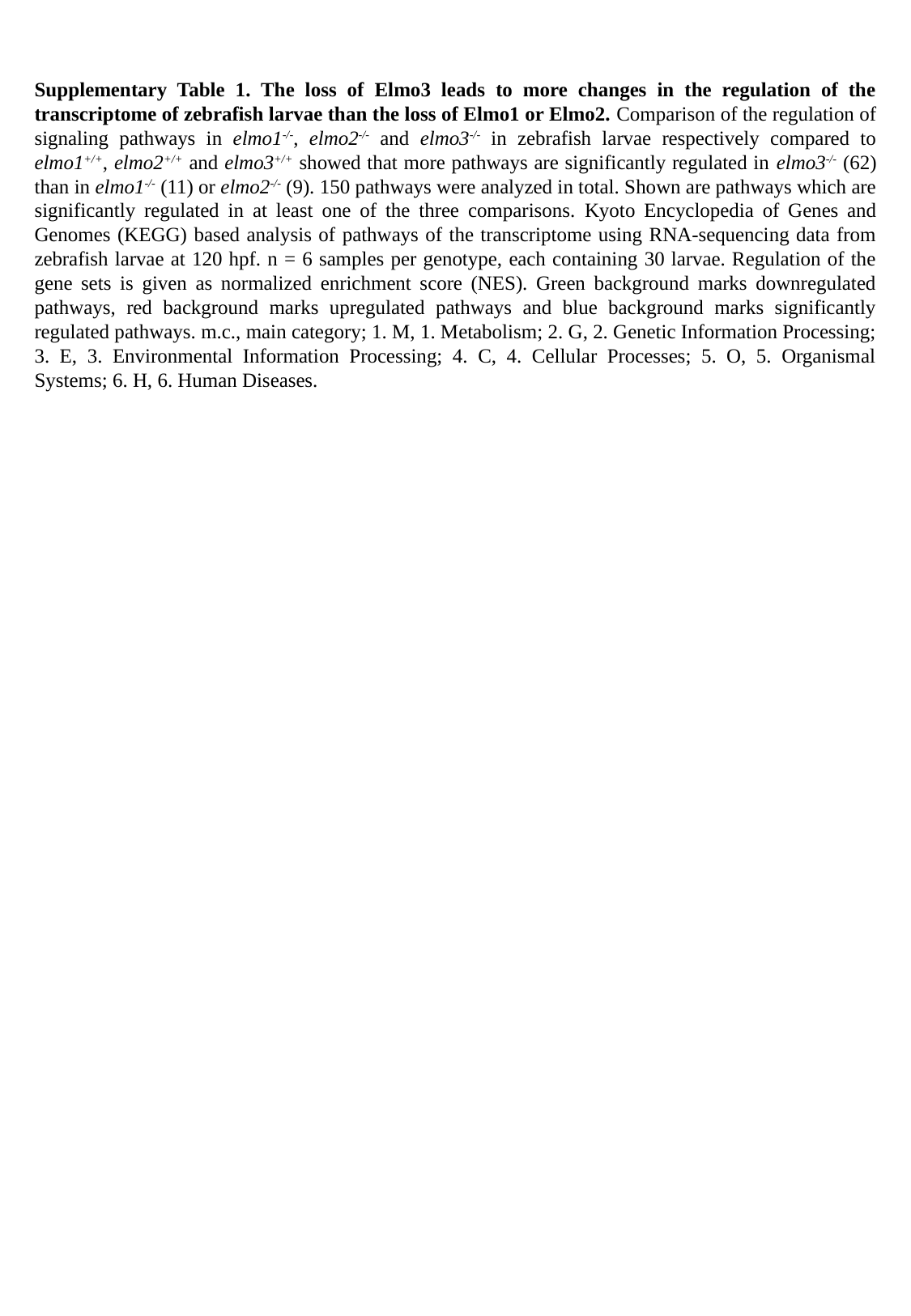

Supplementary Table 1. The loss of Elmo3 leads to more changes in the regulation of the transcriptome of zebrafish larvae than the loss of Elmo1 or Elmo2. Comparison of the regulation of signaling pathways in elmo1-/-, elmo2-/- and elmo3-/- in zebrafish larvae respectively compared to elmo1+/+, elmo2+/+ and elmo3+/+ showed that more pathways are significantly regulated in elmo3-/- (62) than in elmo1-/- (11) or elmo2-/- (9). 150 pathways were analyzed in total. Shown are pathways which are significantly regulated in at least one of the three comparisons. Kyoto Encyclopedia of Genes and Genomes (KEGG) based analysis of pathways of the transcriptome using RNA-sequencing data from zebrafish larvae at 120 hpf. n = 6 samples per genotype, each containing 30 larvae. Regulation of the gene sets is given as normalized enrichment score (NES). Green background marks downregulated pathways, red background marks upregulated pathways and blue background marks significantly regulated pathways. m.c., main category; 1. M, 1. Metabolism; 2. G, 2. Genetic Information Processing; 3. E, 3. Environmental Information Processing; 4. C, 4. Cellular Processes; 5. O, 5. Organismal Systems; 6. H, 6. Human Diseases.

## Slide 12
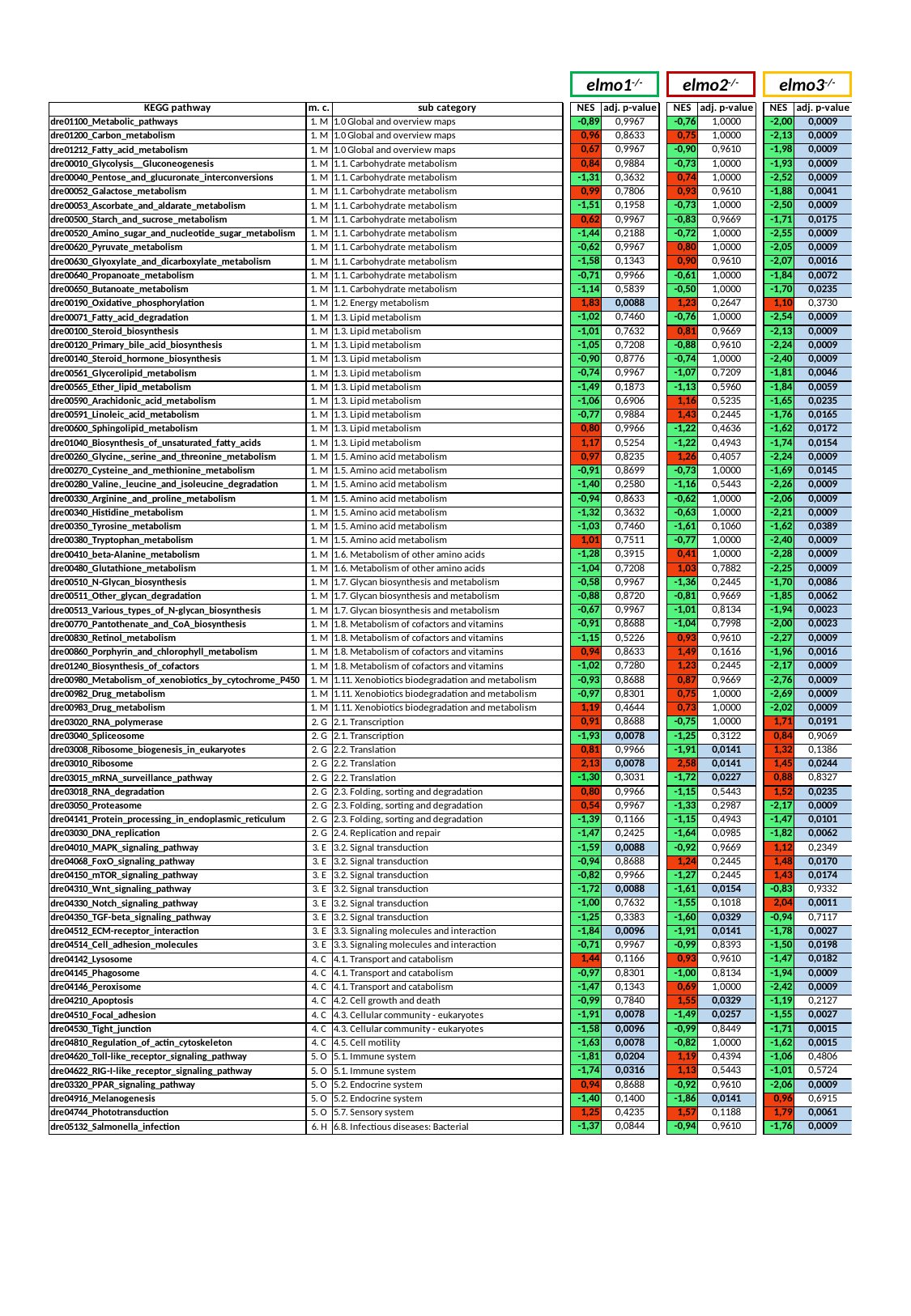

elmo1-/-
elmo2-/-
elmo3-/-

## Slide 13
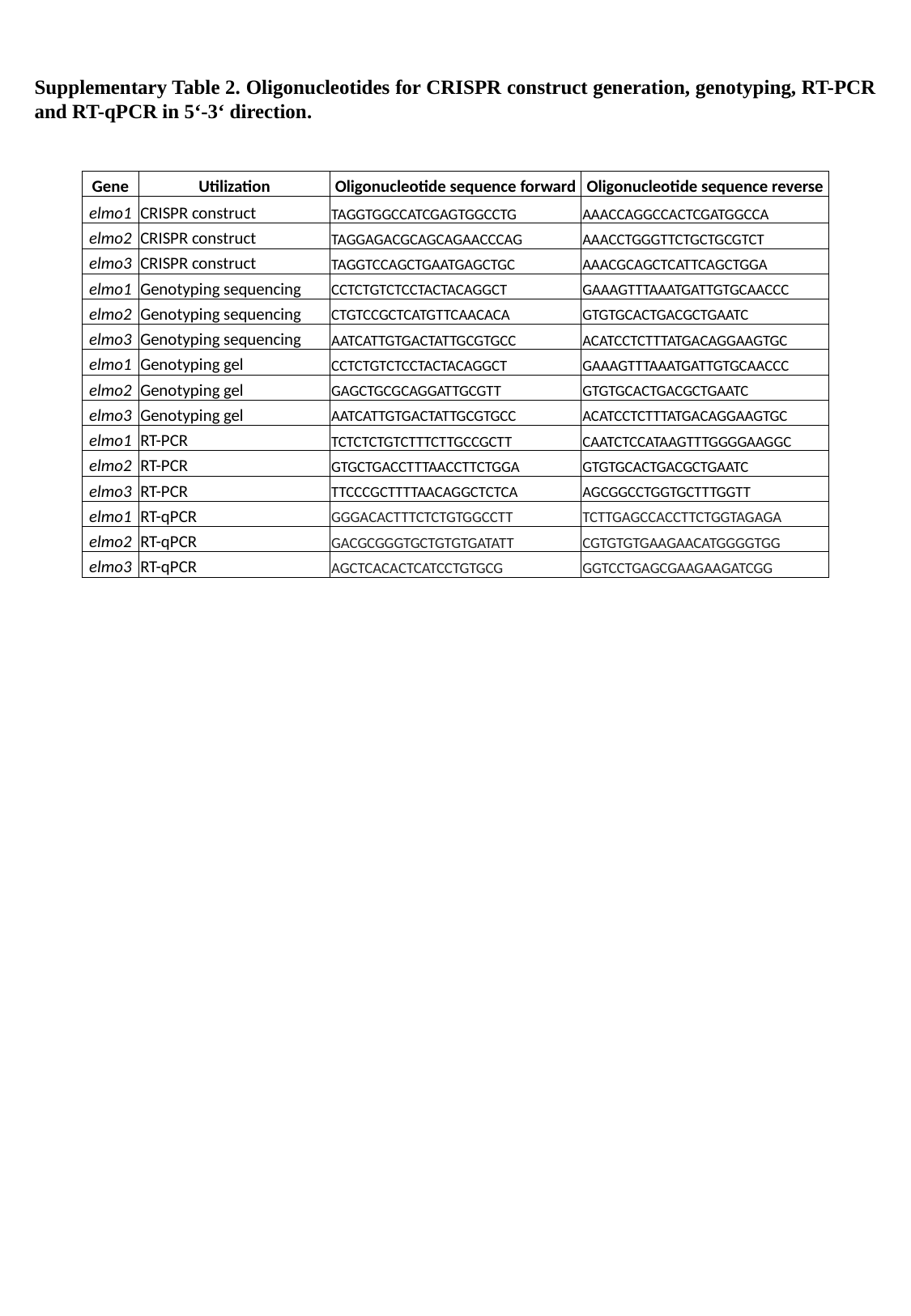

Supplementary Table 2. Oligonucleotides for CRISPR construct generation, genotyping, RT-PCR and RT-qPCR in 5‘-3‘ direction.
| Gene | Utilization | Oligonucleotide sequence forward | Oligonucleotide sequence reverse |
| --- | --- | --- | --- |
| elmo1 | CRISPR construct | TAGGTGGCCATCGAGTGGCCTG | AAACCAGGCCACTCGATGGCCA |
| elmo2 | CRISPR construct | TAGGAGACGCAGCAGAACCCAG | AAACCTGGGTTCTGCTGCGTCT |
| elmo3 | CRISPR construct | TAGGTCCAGCTGAATGAGCTGC | AAACGCAGCTCATTCAGCTGGA |
| elmo1 | Genotyping sequencing | CCTCTGTCTCCTACTACAGGCT | GAAAGTTTAAATGATTGTGCAACCC |
| elmo2 | Genotyping sequencing | CTGTCCGCTCATGTTCAACACA | GTGTGCACTGACGCTGAATC |
| elmo3 | Genotyping sequencing | AATCATTGTGACTATTGCGTGCC | ACATCCTCTTTATGACAGGAAGTGC |
| elmo1 | Genotyping gel | CCTCTGTCTCCTACTACAGGCT | GAAAGTTTAAATGATTGTGCAACCC |
| elmo2 | Genotyping gel | GAGCTGCGCAGGATTGCGTT | GTGTGCACTGACGCTGAATC |
| elmo3 | Genotyping gel | AATCATTGTGACTATTGCGTGCC | ACATCCTCTTTATGACAGGAAGTGC |
| elmo1 | RT-PCR | TCTCTCTGTCTTTCTTGCCGCTT | CAATCTCCATAAGTTTGGGGAAGGC |
| elmo2 | RT-PCR | GTGCTGACCTTTAACCTTCTGGA | GTGTGCACTGACGCTGAATC |
| elmo3 | RT-PCR | TTCCCGCTTTTAACAGGCTCTCA | AGCGGCCTGGTGCTTTGGTT |
| elmo1 | RT-qPCR | GGGACACTTTCTCTGTGGCCTT | TCTTGAGCCACCTTCTGGTAGAGA |
| elmo2 | RT-qPCR | GACGCGGGTGCTGTGTGATATT | CGTGTGTGAAGAACATGGGGTGG |
| elmo3 | RT-qPCR | AGCTCACACTCATCCTGTGCG | GGTCCTGAGCGAAGAAGATCGG |
